# What Is the Impact of Depletion of Immunoregulatory Genes on Wound Healing? A Systematic Review of Preclinical Evidence

**DOI:** 10.1155/2020/8862953

**Published:** 2020-12-07

**Authors:** Bárbara Cristina Félix Nogueira, Artur Kanadani Campos, Raul Santos Alves, Mariáurea Matias Sarandy, Rômulo Dias Novaes, Debora Esposito, Reggiani Vilela Gonçalves

**Affiliations:** ^1^Department of Veterinary Medicine, Federal University of Viçosa, Viçosa, Minas Gerais, Brazil; ^2^Department of General Biology, Federal University of Viçosa, Viçosa, Minas Gerais, Brazil; ^3^Department of Structural Biology, Federal University of Alfenas, Alfenas, Minas Gerais, Brazil; ^4^Department of Animal Science, North Carolina State University, USA

## Abstract

Cytokines and growth factors are known to play an important role in the skin wound closure process; however, in knockout organisms, the levels of these molecules can undergo changes that result in the delay or acceleration of this process. Therefore, we systematically reviewed evidence from preclinical studies about the main immunoregulatory molecules involved in skin repair through the analysis of the main mechanisms involved in the depletion of immunoregulatory genes, and we carried out a critical analysis of the methodological quality of these studies. We searched biomedical databases, and only original studies were analyzed according to the PRISMA guidelines. The included studies were limited to those which used knockout animals and excision or incision wound models without intervention. A total of 27 studies were selected; data for animal models, gene depletion, wound characteristics, and immunoregulatory molecules were evaluated and compared whenever possible. Methodological quality assessments were examined using the ARRIVE and SYRCLE's bias of risk tool. In our review, the extracellular molecules act more negatively in the wound healing process when silenced and the metabolic pathway most affected involved in these processes was TGF-*β*/Smad, and emphasis was given to the importance of the participation of macrophages in TGF-*β* signaling. Besides that, proinflammatory molecules were more evaluated than anti-inflammatory ones, and the main molecules evaluated were, respectively, TGF-*β*1, followed by VEGF, IL-6, TNF-*α*, and IL-1*β*. Overall, most gene depletions delayed wound healing, negatively influenced the concentrations of proinflammatory cytokines, and consequently promoted a decrease of inflammatory cell infiltration, angiogenesis, and collagen deposition, compromising the formation of granulation tissue. The studies presented heterogeneous data and exhibited methodological limitations; therefore, mechanistic and highly controlled studies are required to improve the quality of the evidence.

## 1. Introduction

Cutaneous wounds, according to the World Health Organization (WHO), represent a public problem that affects a major part of the world population and entails elevated costs for health systems. It is estimated that billions of dollars are spent on the acquisition of preventive material and complication treatment every year [1]. About 85% of amputations in diabetic patients are preceded by ulcers, and around 70% of these patients die after five years of amputation [2–4]. Usually, patients with cutaneous wounds present not only pain but also difficulties in everyday activities and loss of function on the affected limb [5]. The efforts to intervene in the wound healing process include ionizing radiation, chemical products, and wound dressings. However, the majority of these efforts have not obtained the desired results, since the pathways through which the skin repair process could be accelerated are still unclear. This is probably why current therapies fail to accelerate wound closure and promote a fast infection-free recovery.

The repair process occurs when the skin is damaged and the connective tissue is exposed [6–8]. The wound healing process is multifaceted and composed of a phase that can be sequential or overlapping [9]. This process is divided into four phases: hemostasis, inflammation, proliferation, and remodeling [6]. These phases involve the action of molecules of cell adhesion, growth factors, and cytokines, besides several other molecules present in the extracellular matrix for it to be efficient [10, 11]. The cytokines and growth factors are important in all phases of the wound healing process. At the beginning of the process, activated platelets release Transforming Growth Factor–*β* (TGF-*β*) and Platelet-Derived Growth Factor (PDGF) resulting in the platelet plugs [12]. Besides that, the degranulation products of the platelet diffused to the extracellular matrix forming a chemotactic gradient of orientation for leukocyte diapedesis [13]. Proinflammatory molecules are released in the inflammatory phase such as interleukins IL-1, IL-2, IL-6, IL-17, and Tumor Necrosis Factor (TNF) that promote the activation of macrophages, neutrophils, and mast cells and stimulate the expression of adhesion molecules [14]. Therefore, macrophages suffer hypertrophy and metabolic increase releasing other growth factors such as TGF-*β*, PDGF, and Vascular endothelial growth factor (VEGF) that are responsible for the activation of endothelial cells and adhesion of molecule sand recruitment of more phagocytes in a cyclical process. Besides, these molecules promote the division and differentiation of keratinocytes and fibroblasts, stimulating the production of collagen early in the repair process [15, 16]. In the proliferative phase, TNF-*α* controls the angiogenesis and formation of the tissue granulation; this is composed of cells and a network of blood vessels responsible for reestablishing regional circulation [17, 18]. The remodeling phase corresponds mainly to changes in the extracellular matrix of the scar tissue, where collagen type III is replaced by collagen type I and occurs the release of mediators such as TGF-*β*, which stimulate the differentiation of myofibroblasts and promote the closure of the wound [19–21]. Also, occurs the release of IL-1, IL-6, and TNF that act through the Nuclear Factor-*κ*B (NF-*κ*B) and signal transducers and activators of transcription-3 (STAT3) pathways, which act to prevent cell death and promote cell proliferation, differentiation, and inflammation [22, 23]. However, many more cytokines and growth factors are present at the wound site, and their dynamic expressions show important temporal and spatial characteristics in the regulation of wound healing processes. Besides that, it is known that important changes in the levels of these molecules affect the production of other cytokines and growth factors [24], and this highlights the complex interactions that occur between these compounds during wound healing. Therefore, these interactions should be considered when interpreting results obtained from the overexpression or elimination of a single immunoregulatory molecule at the wound site.

Currently, the most common and desirable animal model for studying the effect of depletion of specific genes during the repair process are knockout mice [25]. These animal models are important for understanding the role of specific genes in tissue repair, and consequently, for understanding the mechanisms that are involved in the activation of specific cells and the interruption of the repair process [25–29]. Despite this, some questions about the use of this animal model may arise, mainly because this animal is not normally exposed to cytokines and growth factors, and some of these molecules may control more than one cellular activity [25, 30]. However, as it presents the correct ontogenesis process and the normal phenotype, the use of knockout mice is considered successful in the presentation of the physiological and pathophysiological roles of cytokines and growth factors, and it even allows observing the compensation of these molecules activity or the exclusion of receptors excluded by their performance. Therefore, the utilization of genetically altered mice is a powerful method for exploring signal transduction cascades, and it allows more clarification in studies on the impact of single genes in wound healing [25, 30].

It is already known that cytokines and growth factors play an important role in the closure of skin wounds, however, little is known about the design and bias of molecular knockout studies of these small proteins and the impact that this molecular knockout has on skin repair. In addition, typical incisional and excisional wound models in mice are simplistic and at best semiquantitative when used in wound healing analysis. Moreover, a comprehensive analysis of the impact of these molecules that may have proinflammatory and/or anti-inflammatory function over the modulation of important wound healing parameters has never been evaluated by a systematic review. Therefore, we believe that the results of this study will help to understand the main mechanisms involved in the wound healing process and provide a guideline for decision-makers or even researchers in the development of new products and treatments that can accelerate skin wound closure. Based on a detailed analysis of methodological bias, we also evaluated the force of the current evidence by analyzing the advances and limitations of the studies carried out in this field.

## 2. Methods

### 2.1. Focus Question and Registration on the Prospero Platform

This systematic review was based on the following focus question: How can gene depletion related to cytokines and growth factors compromise skin repair? Second, what are the main cytokines and growth factors analyzed in the studies? And what are the main consequences of this depletion? Third, what are the main methodological parameters used to evaluate the evolution of the repair process in the knockout model?

The registration number on the Prospero platform is CRD42020163197.

### 2.2. Bibliographic Search

This systematic review was developed according to the Preferred Reporting Items for Systematic Reviews and Meta-Analysis (PRISMA) guidelines [31], which was used as a guide for the selection, screening, and eligibility of studies. The bibliographic search was performed on September 30, 2019, and was conducted in the following databases: PubMed/Medline (https://www.ncbi.nlm.nih.gov/pubmed), Scopus (https://www.scopus.com/home.uri), and Web of Science (https://www.webofknowledge.com). The descriptors were structured based on search filters built for three domains: (i) animals, (ii) wound healing, and (iii) skin. Inside the animal domain, it was possible to select the knockout animals using this theme (knockout animal) as an eligibility criterion.

The filters on the PubMed/Medline platform were constructed using a hierarchical distribution of the MeSH Terms (Medical Subject Headings) and by the algorithm TIAB (Title and Abstract). These filters were adapted for research in the Scopus platform and Web of Science; however, the filter for animal studies was provided by the Scopus platform (Table S1). The studies were filtered considering the languages: English, Portuguese, and Spanish. Two reviewers (BCFN and RSA) manually searched the reference lists of studies selected in the previous step independently to find additional relevant articles.

### 2.3. Selection of Relevant Studies

After an exhaustive reading of the abstracts, we began to preselect the studies that corresponded to the focus question answers. Studies that were not primary studies, such as brief reports, literature reviews, comments, notes, book chapters, and non-indexed studies, were excluded. Studies with other approaches (i.e., bacteria, virus, radiation, wound suture studies, infected wound, treated wound, wound repair in diabetic mice, or other pathologies) were also excluded. Studies that used only *in vitro* and *ex vivo* studies, knockout for receptor gene, and studies that not evaluated cytokines and growth factors were excluded. Double knockouts were also excluded due to the difficulty of isolating the effect of each gene on cytokines and growth factors and consequently on the wound closure process.

Only studies that met the following eligibility criteria were selected:

(1) *In vivo* studies of skin wound healing with knockout animals whose gene depletion is immunoregulatory molecules

(2) Studies that evaluate changes in cytokines and growth factor expression from the analysis performed on the biopsy

### 2.4. Data Extraction and Management

Three independent researchers (BCFN, MMS, and RVG) selected eligible studies following the analysis of their titles and abstracts. The level of agreement between these reviewers was assessed using Kappa (Kappa = 0.914). When there was doubt, an arbitration was requested from other independent researchers (RDN, RSA, DE, and AKC) to decide whether any given study met the eligibility criteria previously defined, likewise to discard subjectivity in the data collection and selection process, the information was extracted independently and analyzed separately.

The data of the publications were extracted using standardized information such as (1) Publication characteristics and animal models (authorship, country, ethics committee, statistical analysis, lineage, gene depletion, sex, age, and weight); (2) cutaneous wounds (antisepsis, anesthesia, instrument used for biopsy, biopsy collection days, wound area, number of wounds per animal, and wound healing assessment period); and (3) cytokine and growth factor evaluation (cytokine and growth factor analyzed, evaluation methods, and biological material used to evaluate). After this, the data were compared between the reviewers, and the conflict information was corrected. The characteristics that we collected from the studies and used for their evaluation were presented in Table S2. Cytokines and growth factors were classified according to their inflammatory action based on the source cell and the mechanism of action.

### 2.5. Bias Analysis

The quality of the studies was assessed by the criteria described on the SYRCLE's Risk of Bias (RoB) tool (Systematic Review Centre for Laboratory animal Experimentation) [32] and ARRIVE (Animal Research: Reporting of *In Vivo* Experiments) guideline [33]. In relation to SYRCLE's, to facilitate the judgment of scientific articles through the use of characteristics of all studies using animal models, we made questions divided into the following subtopics: Q1-Q3 consider selection bias, Q4-Q5 consider performance bias, Q6-Q7 consider detection bias, Q8 considers attrition bias, Q9 considers reporting bias, and Q10 considers other biases. The articles in the RoB tool were marked with “yes” (low risk of bias), “no” (high risk of bias), or “unclear” (indicating that the item was not reported, and therefore, the risk of bias was unknown). Moreover, we made three additional questions that contributed to the judgment of the studies: Q11: “Was the number of animals per group and the number of animals per cage presented?” We marked “yes” whenever the study mentioned the number of animals per group and per cage. We marked “unclear” for incomplete answers, and “no” whenever nothing was mentioned. Q12: “ What conditions were the animals kept in?” Whenever the answer was yes, we analyzed if the author had mentioned the temperature, humidity, light/dark cycles, water, and food. If the author does not mention these parameters (temperature, humidity, light/dark cycles, water, and food), we stated the study as “unclear.” And whenever this topic was not mentioned at all, we answered “no” for that study. Q13: “Wound closure data were presented with follow-up days, photos, and graphs?” We answered “yes” when the study mentioned the method of wound evaluation in the methodology and presented graphs and photos of wound follow-ups in the results. We answered “unclear” to just some of the information and “no” when nothing was mentioned. The SYRCLE chart was built using Review Manager 5.3 software system. The ARRIVE strategy requires the complete screening of all manuscript sections (abstract to acknowledgments and funding) to evaluate the completeness of scientific reports on animal studies. The screening strategy was based on short descriptions of essential characteristics such as baseline measurements, sample size, animal allocation, randomization, experimental concealment, statistical methods, ethical statement, and generalizability. A table summarizing all relevant and applicable aspects was constructed considering the specificity and aims of the systematic review. The individual adherence to bias criteria and the overall mean adherence were expressed as absolute and relative values [34].

## 3. Results

### 3.1. Characteristics of Publications

**Figure 1:**
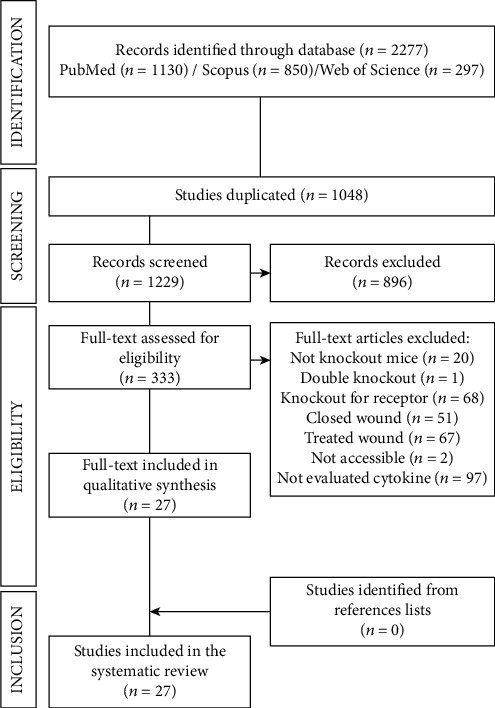
PRISMA diagram. Different phases of the selection of studies for conducting qualitative and quantitative analyses. Flow diagram of the systematic review literature search results. Based on “Preferred Reporting Items for Systematic Reviews and Meta-Analyses: The PRISMA Statement.” http://www.prisma-statement.org. From: Moher D, Liberati A, Tetzlaff J, Altman DG, The PRISMA Group (2009).

The initial research resulted in 1130 studies on the PubMed/Medline, 850 on the Scopus, and 297 on the Web of Science platform, totaling 2277 studies, of which 1048 were excluded because they were duplicates. After reading the titles and abstracts, other 896 studies were excluded, and 333 studies were selected and read in full. Of these studies, only 27 fully met the inclusion criteria and were included in the systematic review (Figure 1).

The studies were conducted in United States of America (25.9%) [27, 35–40]; Japan (18.5%) [26, 41–44]; Germany (11.1%) [45–47]; Brazil, Canada, and China (7.4%, each) [28, 29, 48–51]; and Australia, Singapore, Netherlands, Spain, Taiwan, and Switzerland (3.7%, each) [52–57]. The approval for the use of animals in experimental procedures by the Animal Use and Care Committee was mentioned in 85.2% of the studies, and the statistical analyses performed were specified in 81.5% of the studies.

### 3.2. Characteristics of Experimental Animals

All studies used mice as the animal model and wild type as the control group (100.0%). The animal sex was neglected in 51.9% of the studies, and 33.3% of the studies used males and just 14.8% used females. The age was presented in months and weeks, varying between four and 20 weeks, and 7.4% of the studies neglected this data. The animals' weight ranged from 19 to 30 grams and was reported in two (7.4%) of the studies (Table S2). Only 22.2% [29, 36, 41–43, 51] of the studies allocated the animals individually in cages. Among the strains found in this review, C57BL/6 (29.6%) [27, 35, 43, 46, 47, 51, 54, 55] and BALB/c (14.8%) [26, 41, 56, 57] were the most frequent, besides, 14.8% of the studies [38, 39, 44, 45] omitted this information (Table S2). We found studies with other animal models during the study selection process, such as the rat, but these were discarded due to not meeting the requirements of the other selection criteria.

### 3.3. Characteristics of Wounds

Dorsal skin wounds were made in all 27 studies, 85.2% of studies with excision wounds, and 14.8% with incision wounds (Table S2). There were no similarities in the studies of excisional and incisional wounds in relation to the days when cytokines and growth factors were analyzed. We found other studies that analyzed wounds in other parts of the animal body, but these studies were not considered in this systematic review because they did not meet the requirements of the other selection criteria.

#### 3.3.1. Excision Wounds

Specifications on hygiene and asepsis were reported in 39.1% of the studies, and most of the studies used alcohol as antiseptics (30.4%). Two forms of anesthesia were found in the studies, being ketamine and xylazine as the most common by intraperitoneal injection (26.0%) [27, 45, 46, 48, 55, 56] and isoflurane inhalation as the other compound used (13.0%) [44, 50, 54]. The biopsy punch was the surgical instrument most used to make the wounds (52.2%). Eighteen studies (78.3%) presented data regarding the number and size of wounds, and 21.7% did not provide clear information regarding the number of wounds. The most common size of the wounds was 6 mm (33.3%), and the number of wounds found per animal was two (29.6%). Just 43.5% of studies presented data about the days of wounds biopsy, only 8.7% of the studies [26, 41] carried out the collection of material on 1, 3, 6, 10, and 14 days. The period of evaluation and presentation of data related to wound closure follow-up was presented in 82.6% of studies, and this information was neglected by 17.4% of the studies, as shown in Table S2.

#### 3.3.2. Incision Wounds

Among the studies that evaluated incision wounds, antisepsis, and anesthesia, specifications were reported just in 50.0% [39, 40] of the studies. Regarding the instrument used to perform the wounds, 25.0% of the studies used a scalpel to make the wounds; and this information was omitted in 75.0% of the studies. Half of these studies presented data regarding the number and size of wounds, one study [57] with two wounds per animal, and another study [39] with four wounds per animal; in addition, these studies showed wounds of similar size, 1 cm. Two studies (50.0%) [39, 40] presented the collection days of wounds but there was no similarity. The period of evaluation of wound healing was presented in all studies (100.0%), as shown in Table S2.

### 3.4. Gene Depletion on Wound Healing

Considering the 27 studies evaluated, a total of 30 gene depletions were analyzed. Most of the gene depletion analyzed (60.0%) showed negative effects in the wound healing process, and seven gene depletions showed accelerated wound healing. This probably occurred due to the anti-inflammatory and proinflammatory characteristics of some molecules, and in some cases, the same molecule presented both functions. Moreover, five gene depletions showed no interference in the healing process. The predicted location of the depleted genes was defined based on the location of the corresponding protein, so we found eight genes located exclusively in the extracellular matrix (Interferon-gamma (*IFN-γ*), Granulocyte-Macrophage Colony-Stimulating Factor (*GM-CSF*), Thrombospondin (*TSP1* and *TSP2*), Matrix Metalloproteinase (*MMP9* and *MMP13*), Lumican (*Lum*), and Myostatin (*Mstn*)). Eight genes were located in the intracellular space (Transcription factor NF-E2-related factor 2 (*Nrf2*), Mitogen-Activated Protein Kinase-2 (*MK2*), Serine/threonine kinase (*Akt1*), *Smad3*, Neuronal protein 3.1 (*P311*), Transcription factor proto-oncogene c-Myb (*c-Myb*), Peroxiredoxin 6 (*Prdx6*), and 5-Lipoxygenase (*5-LO*)). Three proteins were located in the plasma membrane (Natural resistance-associated macrophage proteins (*Nramp1*), Connexin 43 (*Cx43*), and Inducible Costimulator (*ICOS*)). Other genes expressed proteins located in different regions of the cells, as in the extracellular matrix and intracellular space (basic Fibroblast Growth Factor (*bFGF*), Interleukins (*IL-6* and *IL-10*), Keratinocyte Growth Factor (*KGF*), and *MMP8*), two were located in the intracellular space and plasma membrane (*MMP14* and Inducible Costimulator Ligand (*ICOSL*)), and one alpha-klotho (*α-kl*) was located in the extracellular matrix and plasma membrane, while one Heme Oxygenase 2 (*HO-2*) was present in these three locations (Table 1).

Among the studies, twenty-one (77.8%) corresponds to the depletion of immunoregulatory genes that influence cytokine and growth factor expression. We can highlight *P311* (TGF-*β* and VEGF); *TSP*: (TGF-*β* and VEGF); *MK2* (GM-CSF, IFN, IL-6, IL-1*β*, and TNF); *ICOS*, and *ICOSL* (IL-6, TNF-*α*, Connective Tissue Growth Factor (CTGF), TGF-*β*, PDGF, VEGF, and IFN-*γ*). Six (22.2%) [26, 35, 38, 41, 46, 51] studies analyzed the depletion of cytokines and growth factors; among them, we can point *IL-6*, *IL-10*, *bFGF*, *IFN-γ*, *KGF*, and *GM-CSF*. Although all the studies included in this review have analyzed the depletion of immunoregulatory genes, just 25.9% studies presented clear data on tests performed to verify the absence of the depleted molecules, since some molecules can be produced by other stimuli (Table 1). Some studies evaluated more than one gene depletion (11.1%) [36, 42, 43], and different studies that analyzed the same depletion (14.8%), for example, two studies analyzed the effects of the *TSP2* depletion [36, 37] and two studies analyzed the effects of the *P311* depletion [27, 28].

Thirteen (48.1%) studies were related to gene depletion that influenced the organization and deposition of collagen fibers; among them, 29.6% of the studies showed a reduction of collagen fibers at the wound site, 14.8% of the studies showed an increase in collagen deposition, and 7.4% presented results similar to wild type (Table 1). Fourteen studies (51.8%) presented clear data on the presence of inflammatory cells in the wound area, being that eight studies presented a reduction, four presented similar results to wild type, and just two presented an increase in these cells. Another mechanism that has been thoroughly analyzed was angiogenesis and vascularization. Six (22.3%) studies presented reduction for these important parameters in the wound healing process, four (14.8%) presented an increase, and two (7.4%) did not present alterations on these markers (Table 1).

Furthermore, some individual study results were somewhat conflicting, for example, *P311* gene depletion, in which one study showed that the depletion of this gene resulted in delayed wound closure, while in the others, the changes were not significant when compared to the wild type. In addition, these results demonstrated that direct gene depletion of immunoregulatory molecules that indirectly control the cytokines and growth factors can influence one specific or several cellular pathways and consequently proinflammatory and anti-inflammatory molecules reflecting a delay or acceleration of the wound healing process (Table 1). Therefore, we observed that the depletion of some genes delays the healing process because it promotes a decrease in cellular activity in the inflammatory, proliferative, and remodeling phases. On the other hand, the gene depletions related to improvements in the repair process are mainly related to the proliferation and remodeling phases of skin repair.

### 3.5. Main Characteristics and Methodologies Applied in the Investigation of Immunoregulatory Molecules in Wound Healing

**Figure 2:**
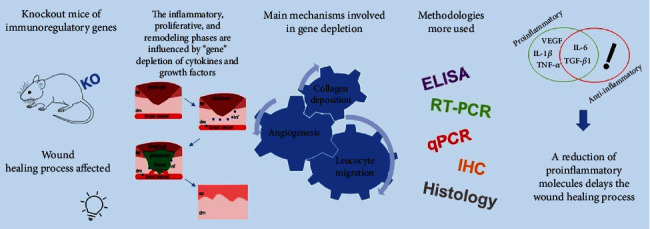
A schematic diagram showing the influence of different immunoregulatory genes of knockout mice on the wound healing process, the main phases affected by genetic silencing, and consequently the main mechanisms involved in this process, and the most common techniques used for this type of analysis. KO: Knockout; ELISA: Enzyme-Linked Immunosorbent Assay; RT-PCR: Reverse Transcription Polymerase Chain Reaction; qPCR: Real-Time quantitative Polymerase Chain Reaction; IHC: Immunohistochemistry; TGF-*β*1: Transforming Growth Factor beta 1; VEGF: Vascular Endothelial Growth Factor; TNF-*α*: Tumor Necrosis Factor-alpha; IL-6: Interleukin-6; IL-1*β*: Interleukin-1 beta; ep: epidermis; dm: dermis; inf: inflammatory molecules.

All studies selected in this systematic review used wound tissue for cytokines and growth factors evaluation, and the predominant evaluation methods were Enzyme-Linked Immunosorbent Assay (ELISA) (29.6%), followed by Reverse Transcription Polymerase Chain Reaction (RT-PCR) (18.5%) (Table S2). The materials extracted from the wound tissues used for these analyses were RNA (55.5%) and protein (37.0%) (Table S2 and Figure 2).

#### 3.5.1. Cytokines and Growth Factors Analyzed

The gene depletion using exclusively proinflammatory molecules was done in 22.2% of the studies, we can highlight IL-1*α*, IL-1*β*, IL-12, IL-18, GM-CSF, Acid Fibroblastic Growth Factor (aFGF), Basic Fibroblastic Growth Factor (bFGF), Epidermal Growth Factor (EGF), CTGF, PDGF, VEGF, VEGF-A, TNF, TNF-*α*, and TGF-*β*2. Most of the studies (81.4%) analyzed molecules with both functions (IL-6, Angiopoietin (Ang-2), IFN- *γ*, TGF- *β*, and TGF-*β*1). Only four studies (14.8%) analyzed the effect of gene depletion exclusively in anti-inflammatory molecules (IL-4, IL-10, Ang-1, and TGF-*β*3) (Table 1).

Among the most studied cytokines and growth factors, we can highlight TGF-*β*1 (40.7%), followed by VEGF (33.3%), IL-6 (18.5%), TNF-*α* (18.5%), and IL-1*β* (14.8%) (Figure 2). These molecules are involved in the different phases of the wound healing process, such as inflammation, cell migration, granulation tissue synthesis, and reorganization and synthesis of the Extracellular Matrix (ECM), and reepithelization. Seven studies (25.92%) showed reductions in these molecules, consequently, a delay in wound closure and a decrease in the wound healing process (Table 1). Another important studied molecule was IFN-*γ* (11.1%), whose direct depletion promoted an accelerated wound healing, with increased angiogenesis and collagen deposition, but curiously, when IFN-*γ* was indirectly evaluated as the result of the depletion of other genes, for example, ICOS and ICOSL, the reduction of these molecules contributed to delaying the wound healing process. These findings indicate that the depletion mechanisms are complex, able to interfere with various cytokines and growth factors, and capable of acting on several pathways, and this reflected on the phases of the wound healing process.

Among the anti-inflammatory cytokines studied, we can highlight gene depletion related to IL-10 cytokine (11.1%) [29, 43, 46]. The studies showed that the reduced expression of IL-10 promoted a fast-wound closure and decreased granulation tissue formation and proliferation of the myofibroblasts, neutrophils, and macrophages. However, only one study (3.7%) [46] demonstrated that this cytokine was depleted in the tissue, which increased angiogenesis, high myofibroblast differentiation, macrophage infiltration, and collagen deposition (Table 1), reinforcing the role that anti-inflammatory modulation molecules play on the inflammatory phase during skin wound.

### 3.6. Other Analyses

In general, studies identified in this review support the evidence that the depletion of immunoregulatory genes did affect the wound healing process. Although all studies have performed histological and quantification of cytokine in the wound tissues, some studies have also performed other techniques, some of which have caught our attention due to the relevance of this process. One study [45] (3.7%) performed oxidative analyses and observed that the knockout of *Prdx6* left endothelial cells more sensitive to the occurrence of oxidative stress during inflammation resulting in severe hemorrhage in the granulation tissue. Two studies (7.4%) [26, 41] performed Myeloperoxidase (MPO) assay to assess neutrophil infiltration and observed lower MPO activity and neutrophil infiltration in the knockout mice. Four studies (14.8%) [29, 36, 51, 54] performed chemokines analyses and observed the reduction of Monocyte Chemoattractant Protein (MCP-1) and Macrophage Inflammatory Protein (MIP-1 and MIP-2) in the knockout mice. These findings showed that markers of oxidative stress are important tools in evaluating the redox balance of healthy and damaged tissues, mainly in the inflammatory phase, indicating in general, damage in tissue, and a delay in wound closure.

### 3.7. Risk of Bias and Methodological Quality Assessments

**Figure 3:**
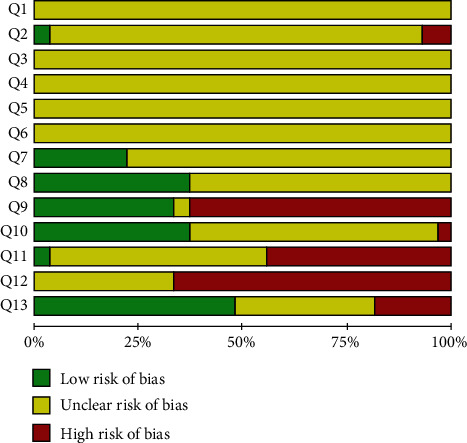
Bias risk results and methodological quality indicators for all studies included in this systematic review that evaluated the effect of gene depletion on excisional and incisional wounds. Q1: Was the allocation sequence adequately generated and applied? Q2: Were the groups similar at baseline or were they adjusted for confounders in the analysis? Q3: Was the allocation to the different groups adequately concealed? Q4: Were the animals randomly housed during the experiment? Q5: Were the caregivers and/or investigators blinded from knowledge regarding which intervention each animal received during the experiment? Q6: Were animals selected at random for outcome assessment? Q7: Was the outcome assessor-blinded? Q8: Were incomplete outcome data adequately addressed? Q9: Are reports of the study free of selective outcome reporting? Q10: Was the study free of other problems that could result in a high bias risk? Q11: Was the number of animals per group and number of animals per cage presented? Q12: What conditions were the animals kept in? and Q13: Wound closure data were presented with follow-up days, photos, and graphs?

**Figure 4:**
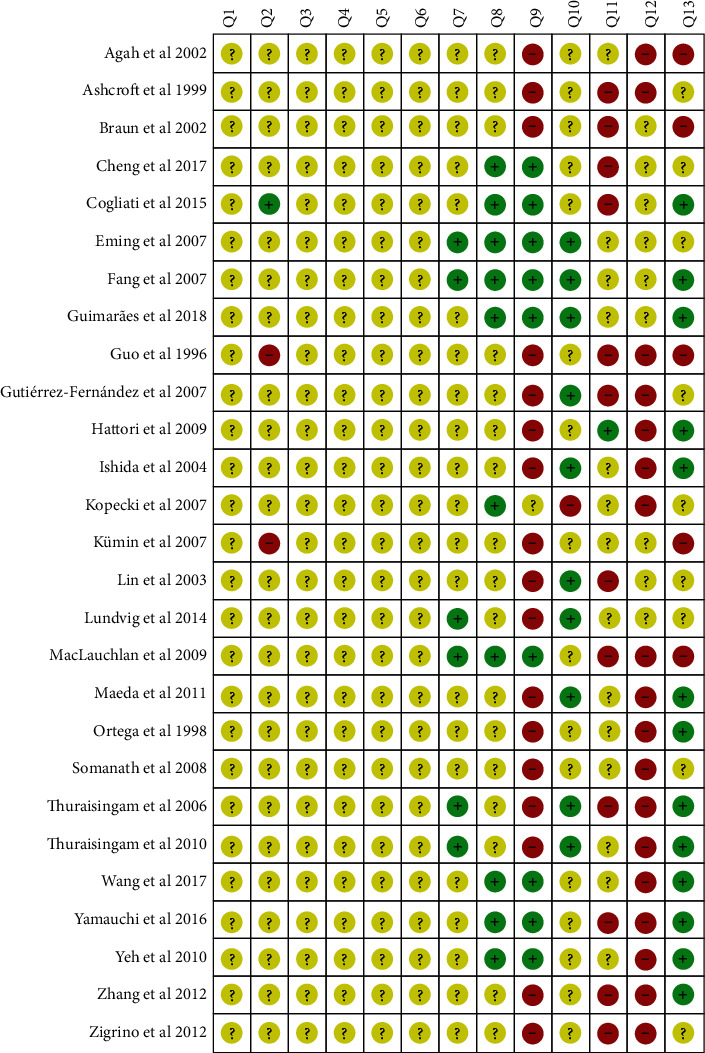
Risk of bias summary: review authors' judgments about the risk of bias items for each included study. Green: low risk of bias; Yellow: unclear risk of bias; and Red: high risk of bias.

**Figure 5:**
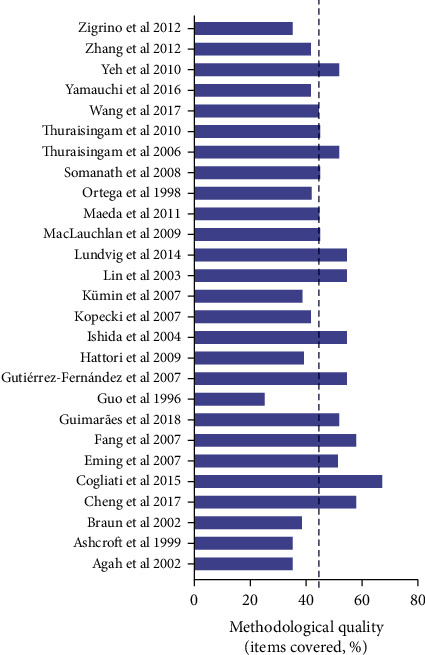
Analysis of methodological bias (reporting quality) for each study included in the review. Based on Animal Research: Reporting of In Vivo Experiments (ARRIVE) guidelines (http://www.nc3rs.org.uk/arrive-guidelines). The dotted line indicated the mean quality score (%). Detailed bias analysis stratified by domains and items evaluated is presented in Table S3.

The reporting bias based on SYRCLE analysis was detailed in Figures 3 and 4. None of the studies fulfilled all methodological criteria of bias risk (100.0%). The sequence generation process (Q1) was not reported in any of the studies (100.0%). The similarity of animal characteristics to each other (Q2) was not reported clearly in 26 studies (96.3%). The information about allocation concealment (Q3), random housing (Q4), blinding of caregivers (Q5), and random outcome assessment for detection bias (Q6) were not clear in any of the studies (100.0%). Moreover, the outcome assessor was not reported to have been blinded (Q7) in 21 studies (77.7%). Incomplete outcome data (Q8) were shown in 17 studies (62.9%). Nine studies (33.3%) presented a high risk for reporting bias (Q9), and ten studies (37.0%) did not present other potential sources of bias (Q10). Three other quality indicators were used to assess the methodological quality of the studies, just 3.7% of the studies reported the distribution of animals by group and cage (Q11), nine (33.3%) studies presented unclear data about animal conditions (Q12), and 51.8% of studies presented clear data on wound closure follow-up (Q13). The results of the ARRIVE analysis show that the most recent studies have better met the methodological quality criteria analyzed (Table S3 and Figure 5). None of the studies fulfilled all methodological criteria, and the mean quality score of all studies reviewed was 46.3 ± 9.1. No study reported the number of animals per group, blinding and randomization of the experiment, total number of animals, how to choose the sample size and repetitions, complete details of how the animals were allocated to the experimental groups, or the health status of the animals. Most studies presented analysis results carried out with precision measurements (96.3%), data on authorizations for use of animals (85.2%), and statistical analyses (81.5%). The characteristics of the animals that were most mentioned were the age (92.6%) followed by the strain (85.2%). Anesthesia (63.0%) was more addressed among the important items of surgical procedures, and the absence of pathogens (37.0%) was the most mentioned characteristic about accommodation place.

## 4. Discussion

### 4.1. General Characteristics of the Studies

In our study, we conducted a systematic review to investigate the direct influence of gene depletion of immunoregulatory molecules in the wound healing process. In addition, we analyzed the methodological quality of the studies that address this theme. We found 27 studies that met all the selection criteria. Optimism in this field has been tempered by some limitations of this animal model usage in wound healing studies; mainly, they required a substantial amount of the resources in terms of time and expense to be developed. This type of research requires laboratories with sufficient technology to identify changes in the cytokines and growth factors profile, and this condition is easily found in economically strong countries. Moreover, there are high costs for acquiring knockout animals since few laboratories are equipped for generating to create their knockouts. These points may justify our findings for the predominance of the studies in countries such as the United States of America, Germany, and Japan. Another interesting result about characteristics in the studies was that the gene depletion of the proinflammatory molecules was preferably analyzed when compared to that of the anti-inflammatory molecules. Probably, this has occurred because there is no consensus about the time-independent and concentration-dependent responses to individual proinflammatory cytokines. Furthermore, proinflammatory molecules are involved in the initial phase of the cutaneous healing process and are important to modulate all phases of this process. Within these phases, we can highlight the following points: hemostasis phase, characterized by the clearing of wound debris by inflammatory cells, and it occurs during the initial response. Granulation tissue formation and angiogenesis start the generation of a new provisional wound matrix. Both extracellular matrix synthesis and its appropriate degradation are necessary, especially during proliferation and remodeling. Therefore, the depletion of these molecules can compromise the whole healing process.

### 4.2. Animal Characteristics

Mice were the main animal model used for the study of gene depletion on the wound healing process. These results were expected since mice share many genes with humans, and consequently, knockout mice give crucial information that can be used to better understand this disease in humans. Additionally, rats and mice are popular experimental models because of their low cost, availability, and ease of care and handling, allowing researchers to use a relatively large number of animals for their experiments, thereby generating a greater degree of reliability in the results [58–65]. On the other hand, knockout mice also offer a biological context in which drugs and other therapies can be developed and tested [66]. Therefore, knockout mice still offer one of the most powerful means today for studying gene functions in a living animal. About the wound healing process, mice are organisms applied in studies for wound healing because this model allows the histological monitoring of the process [67]. Moreover, this model allows the realization of macroscopic, biochemical, and biomechanical measurements [68]. Despite the advantages of mice models, there are also some negative points to the use of this animal model, such as skin characteristics. Rodents are loose-skinned animals with a panniculus carnosus layer and lack well-developed deep attachments to the skin compromising the study of important phases of the wound healing process, like granulation tissue formation and reepithelization. However, the positive points outweigh the negative ones for this model because mice, rats, and humans exhibit the same stages of wound healing, with immunoinflammatory and microstructural convergences, based mainly on similar profiles of regulatory molecules (i.e., cytokines and growth factors) and composition of extracellular matrix (i.e., glycosaminoglycan's, collagen, and noncollagen proteins).

Most studies founded in this review did not provide sufficient information on methodology development. For example, most of the studies did not present data regarding the sex of the animals although this is a very important piece of information since studies using females are subject to more variation due to their hormonal changes [67]. Another interesting point is related to age, although some variations were reported, the experiments used animals within similar age ranges; this is an important feature of the studies that evaluate wound healing because the aging of mice influences wound closure, reepithelization, and filling of granulation tissue [69]. Surprisingly, only six studies mention that the mice were housed individually in cages. This is an important point because when placed in individual cages, there is no interference from the saliva of other animals in the wound closure process, and also, there is a reduction of injury risk and aggravation of the wound under analysis [67]. As shown, the lack of information about animals and their care, or even the neglect of certain characteristics of the experimental design, can compromise the results, thus increasing the bias of the studies, and consequently, reducing the reliability of the results found.

### 4.3. Wound Healing Characteristics

Considering the characteristics of the wound, most studies used an excisional wound, a model that generally presents high precision and few variations for wound healing evaluation, so in general, this model is efficient in comparing wild type and genetically modified animals [70]. Besides that, another important parameter that must be analyzed is the spatial position of the wounds because the choice of the proper location allows minimizing differences and interferences, mainly about tensile strength and skin tissue resistance [67]. In our revision, all studies made dorsal wounds on the mice. Probably, this location was chosen because, in this location, the mice cannot lick the lesion area, which could interfere with the reliability of the results, since it has been confirmed that licking behavior can promote wound healing because the salivary gland is a reservoir for many growth factors in rodents [71]. Furthermore, the dorse is an appropriate area to perform a biopsy because it is easy to manipulate and allows obtainment of a sufficient quantity of tissues to study the contraction index of wound and reepithelization rates, and consequently, total tissue repair [72].

Among the surgical instruments used to perform skin excision biopsies, the punch was the most used, possibly due to this instrument presenting defined measures, and thus, ensuring that all wounds are the same size. As mentioned earlier, the evaluation periods of the wound healing process were different for excision and incision models, this must-have occurred because the models have distinct characteristics. Incision wounds heal by primary intention, and excision wounds heal by second intention [73]. Moreover, we observed that the biopsy collections for histological analysis and immunoregulatory molecules did were not performed daily to accompany the wound healing process. This probably occurred because performing biopsies every day may be impracticable due to a large number of animals and to the fact that the wound healing phases occur in several phases for several days. Although the repair phases are not mutually exclusive but overlapped over time. It is possible to estimate the days for each phase. Approximately, the inflammatory phase will occur from day 1 to day 3, the proliferative phase from day 2 to day 14, and the remodeling phase would start on day 13 [74]. Based on this, the researchers can select the best moments to perform the analyses to attend all phases of the wound repair, since the moment of the analyses are critical and must include clear information not only for macroscopic closure but also for histological analysis and immunoregulatory molecules.

### 4.4. Analysis of Cytokines and Growth Factors in Skin Wound Healing Process

Cytokines and growth factors are considered biomarkers that make it possible to understand the progression of repair processes because, in high concentrations, they indicate activation of inflammation and proliferation-related pathways, which is very important for the wound healing process [75, 76]. Currently, several tests allow measuring the concentration of cytokines and growth factors, being ELISA tests and PCR technologies widely used. However, ELISA has limitations as it does not provide information on the biological potency of the detected proteins and fails to provide information to indicate the identities and frequencies of the individual cytokine and growth factor producing cells [77]. Whereas the RT-PCR, which was the most used PCR type in the studies found in this review, use the specific mRNA expression of the molecule evaluated for the analysis of cytokines for the minimum amount of molecules released by cells [78]. This allows the quantitative comparison of these molecules in different targets [79]. This test is widely used for this type of analysis, and its execution is considered as quick and easy, although its results may differ from those found by the ELISA test and do not provide exact information on the concentration of cytokines [78]. Thus, it is necessary that when performing the analysis of cytokines, the person in charge evaluates the available methodologies and chooses the one that meets their demands with a greater degree of sensitivity to obtain reliable data.

The important role that cytokines and growth factors play in the wound healing process is already known. However, this systematic review is the first to investigate and compare the most studied cytokines and growth factors as well as their behavior in different pathways. Our findings showed that the depletion of the most studied immunoregulatory genes was TGF-*β*1 followed by VEGF. TGF-*β*1 has important functions in the wound healing process mainly in the regulation of inflammation and also in the accumulation of collagen and resistance of ECM [80]. Additionally, VEGF has played an important role during the skin repair process, as it stimulates angiogenesis, which is essential for the transport of oxygen and nutrients to the wound site. It influences the recruitment of inflammatory cells, granulation tissue formation, fibroblasts proliferation, and remodeling tissue [81]. Overall, the depletion of these genes negatively influenced the production of other proinflammatory cytokines and consequently promoted the decrease of inflammatory cell infiltration, angiogenesis, and collagen deposition. These genes are important in the regulation of keratinocytes and fibroblasts proliferation and consequently on the recovery of the skin tissue which justifies the fact that most studies evaluated the action of these molecules in the cutaneous lesions. Interestingly, there were conflicting results when other genes were analyzed, for example, our findings indicated that the reduction or silencing of proinflammatory cytokines and growth factors like GM-CSF, bFGF, and IL-1*β* showed a negative impact on the skin repair process. These findings corroborate with previous evidence that GM-CSF influences important phases of the repair process because it promotes neovascularization and collagen deposition and contributes to the composition of the vascular collagenous matrix [81], and consequently, represents important markers to understand the main proinflammatory mechanisms involved in the wound healing process.

Other constantly studied molecules were the interleukins, and the signaling pathway IL-1*β* cytokine was one of the most frequently analyzed in studies, and its action is related to the activation of the pathway of NF-*κ*B and Mitogen-activated protein kinases (MAPK) [82–84]. Both survival and proliferation pathways promote the activation of Toll-like receptors, which contribute to the activation and regulation of the immune response [85]. The NF-*κ*B corresponds to a family of inducible transcription factors, which regulates genes involved in the immune and inflammatory responses [86]. It induces the expression of various proinflammatory genes, like cytokines, and regulates the survival, activation, and differentiation of inflammatory and innate immune cells [87]. The MK2 is a serine/threonine kinase of the p38 MAPK pathway, and it was a genic depletion studied in this review. This depletion resulted in delayed wound healing, a decrease of angiogenesis, and collagen deposition. This probably occurs because the MK2 phosphorylation is important for cell cycle regulation, acting on remodeling, cell development and migration, and cytokine production [88–91], and in their absence, these processes can be affected. The IFN-*γ* is an important cytokine in the activation of pathways involved with the cell cycle; interestingly, this depletion promoted accelerated wound healing. The interferon family is divided into three types of IFN, with IFN-*γ* being a cytokine involved in the adaptive immune response. These cytokines have been playing a key role in the activation of proinflammatory macrophages [92, 93] and act with anti-inflammatory function modulating proinflammatory cytokines and apoptosis [94].

In our review, the lack of studies and analyses performed with cytokines and growth factors exclusively anti-inflammatory functions was evident, and this calls into question the accuracy of the presentation of the effect of these molecules in the wound healing process. However, gene depletion of IL-10 showed beneficial effects for the repair process with increased epithelization, macrophages infiltration, angiogenesis, collagen production, and myofibroblasts differentiation. Besides, the depletion of these cytokines stimulated the expression of other cytokines such as VEGF-A, important for skin wound healing. In addition, IL-10 acts as balancing cellular signaling by inhibiting proinflammatory cytokines, inhibiting the antigen presentation by dendritic cells, or inhibiting macrophage activation and infiltration into the site of the wound [95]. Another interesting point was that few studies in this review have evaluated cytokine isoforms and growth factors such as three isoforms of TGF-*β* (TGF-*β*1, TGF-*β*2, and TGF-*β*3) [27], isoform VEGF-A [46], and isoforms Ang-1 and Ang-2 [53]. However, the study of different isoform assessment whenever possible is important because these may be antagonistic to each other, have different functions, and applications in the wound healing process. Based on these findings, we see the importance of evaluating cytokines and growth factors, including their isoforms, since they have important functions for each phase of the wound healing process and act in different pathways in the entire process.

### 4.5. Other Analyses

Analyses of oxidative stress are very relevant when we study the wound closure process because inflammation occurs in skin lesions, thus macrophages produce free radicals through the respiratory burst process, such as Reactive Oxygen Species (ROS) that promote changes in lipids, proteins, and cellular DNA impairing cellular longevity [96, 97]. As mentioned previously, in some studies, we found the oxidative analysis of wound tissues and analyses associated with free radicals in these same tissues; however, in some cases, the participation of the molecules analyzed in the oxidative processes is not clear. The MPO is an enzyme that catalyzes the formation of ROS because it reacts with hydrogen peroxide forming free radicals, which results in oxidative damage to the tissue in case of imbalance with antioxidant enzymes [98–100]. While MCP-1, MIP-1, and MIP-2 are chemokines related to inflammation. MCP-1 in high concentrations generates respiratory explosion and consequently the release of ROS [101]. MIP-1 and MIP-2 are produced by various inflammatory cells, recruit and activate neutrophils [102], and these produce ROS which under normal conditions, eliminate damaged tissue, but in excess cause tissue injuries [103].

As seen, there are several possibilities to estimate the occurrence of oxidizing activity in tissues, and these analyses are extremely important to rule out factors that interfere with the wound healing process, as it is known that the excessive production of ROS impairs healing [97].

### 4.6. Synthesis of the Mechanistic Theory of Studies

**Figure 6:**
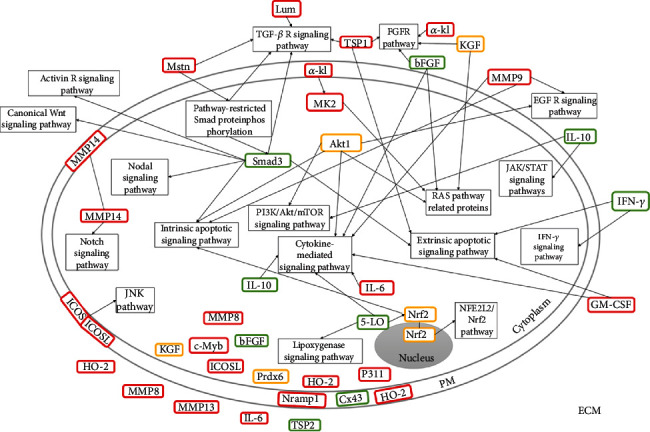
Location of the molecules of depleted genes addressed in this review and their participation in metabolic pathways involved in the wound repair. The effect of depleted genes on wound closure was shown by the colors: red (delayed), green (accelerated), and yellow (unchanged). PM: plasma membrane; ECM: extracellular matrix; 5-LO: 5-Lipoxygenase; *α*-kl: alpha-Klotho; bFGF: basic Fibroblastic Growth Factor; Cx43: Connexin 43; GM-CSF: Granulocyte-Macrophage Colony-Stimulating Factor; HO-2: Heme Oxygenase 2; ICOS: Inducible Costimulator; ICOSL: Inducible Costimulator Ligand; IFN-*γ*: Interferon-gamma; (IL-10, IL-6): Interleukins; KGF: Keratinocyte Growth Factor; Lum: Lumican; (MMP8, MMP9, MMP13, MMP14): Matrix Metalloproteinase; MK2: Mitogen-Activated Protein Kinase-2; Mstn: Myostatin; Nramp: Natural resistance-associated macrophage proteins; P311: Neuronal protein 3.1; Prdx6: Peroxiredoxin 6; Akt1: Serine/threonine kinase; (TPS1, TPS2): Thrombospondin; Nrf2: Transcription factor NF-E2-related factor 2; c-Myb: Transcription factor proto-oncogene c-Myb. Signaling pathways: Activin R: Activin Receptor; FGF R: Fibroblast Growth Factor Receptor; EGF R: Epidermal Growth Factor Receptor; TGF-*β* R: Transforming Growth Factor-*β* Receptor; NFE2L2/Nrf2: Nuclear Factor, Erythroid 2 Like 2; JNK: c-Jun N-terminal kinase; IFN-*γ*: Interferon-gamma; JAK/STAT: Janus Kinase Signal Transducer and Activator of Transcription.

Although all studies have analyzed the influence of the depletion of immunoregulatory genes on the wound healing process, not all of them make clear the main mechanisms involved in this process after specific gene depletions, even though this information is important to justify the analysis. In addition, it is important to consider that the depletion of a gene can generate a chain reaction and impair the entire repair metabolic process [52]. However, our findings showed that there were three points based on the location of the corresponding protein that directly interferes in the wound healing process, which are (1) extracellular molecules; (2) intracellular molecules; and (3) membrane molecules. Although all molecular locations have distinct and important functions in cellular processes, we can see that extracellular molecules act more negatively in skin wound healing when silenced (Figure 6), probably because they act as signals for several important metabolic pathways. In addition, some molecules that have different locations may do different functions depending on their location and not participate in repair-related metabolic pathways, for example, bFGF in the extracellular space acts on metabolic pathways that result in cell proliferation, migration, differentiation, and apoptosis, while in the intracellular environment, this molecule acts on antiviral immunity [104].

As the studies are quite heterogeneous and the type of gene depletions is different in most of the studies, it has been difficult to determine the real impact of depletion of immunoregulatory genes on wound healing. However, we observed that extracellular molecules act more negatively in the wound healing process when silenced, and the TGF-*β* family of growth factors has been addressed frequently in studies and mechanistic theories, which suggests that this molecule is very important for the wound healing process, mainly due to its participation in the TGF-*β*/Smad pathway that has so many other linked metabolic pathways. TGF-*β*/Smad signaling pathway is involved in the regulation of proliferation, differentiation, and survival or apoptosis of many cells. In addition, Smads are phosphoproteins, which when phosphorylated start signaling the TGF-*β* family, which results in a cascade of protein-protein and protein-DNA interactions [105]. Macrophages and TGF-*β*1 were frequently mentioned in explaining these processes, this is probably because macrophages play an important role in the repair process, since they act by secreting growth factors like TGF-*β* and inflammatory mediators important for collagen deposition, wound contraction, and angiogenesis, while TGF-*β*1 performs functions at all phases of the repair process, is essential for regulating collagen deposition [106], and can stimulate VEGF transcription in various cells [80, 107]. In addition, there are reports of changes in TGF-*β* levels of different forms, either by kidnapping by competition for the receptor or by sequestering TGF-*β* by other molecules, as Decorin [108].

Although the other metabolic pathways were less affected (Figure 6), they are also important for the occurrence of the repair process and can act in several biological processes, such as inflammation, proliferation, remodeling, growth and cell differentiation, on the immune system, and tissue repair [109–112]. We also found molecules related to pathways that result in apoptosis, such as extrinsic and intrinsic apoptotic signaling pathways. The activation of these pathways results in a cascade of proteases, the caspases, resulting in cell death [113] and acts in removing unwanted cells [114]. In addition to the pathways that act directly on the wound healing process, we had depleted genes that are involved in pathways related to ROS, such as 5-LO in Lipoxygenase signaling pathway and Nrf2 in Nuclear factor, erythroid 2 like 2 (NFE2L2/Nrf2) signaling pathway. Even 5-LO can activate Nrf2 which has a cytoprotective effect [115, 116]. Some molecules, such as matrix metalloproteinases, were not linked to metabolic pathways involved in the wound healing process, but they play an extremely important role in this process, their silencing results in prolonged inflammation and delayed wound closure.

## 5. Methodological Quality of the Animal Studies

Genetic advancements in recent years have made mouse models of human disease processes increasingly popular considering their numerous other advantages. However, no systematic review has been reported to investigate the impact of depletion of immunoregulatory genes on wound healing. The main strength of this study is its novelty and the applied findings that can be useful to provide a direction for future studies in this field and the development of decision-making for therapeutic alternatives. Also, the metabolic network description of this study may help to understand the main mechanisms involved in the alterations triggered by gene depletion and, consequently, the translation to the human health assessment. Therefore, systematic reviews are essential tools for summarizing evidence accurately and reliably, assisting risk assessment, and providing evidence of the benefits of health-related interventions [117].

This review also has some limitations. The bias analysis demonstrated that fundamental characteristics, such as random sequence generation or random outcome assessment and blinding of participants (caregivers and outcome assessor), were not reported in the studies. In addition, some records provided incomplete outcome data and insufficient information, which affect the accuracy of the results. Overall, the evidence of the individual studies showed wide heterogeneity and so it was not possible to compare the data statistically. This kind of comparison should be avoided because it generates evidence that presupposes an apparent external validity (generalizability), which is not supported by the available data set. In this sense, we identified that each study presented marked differences regarding the experimental model and methods of data collection, analysis, and interpretation, as well as biopsy procedures, wound monitoring, evaluation of immunoregulatory molecules, and mechanisms involved in the healing process contributing to the increased risk of bias. In individual studies, each element of methodological bias is associated with some degree of variability in the research outcomes, with a direct impact on the quality of evidence. However, it is important to emphasize that all types of reviews have limitations, and these limitations are more evident in systematic review studies once flaws in methodological and incomplete reports can produce inaccurate and unreliable conclusions. In our case, the major limitation was the heterogeneity of the studies, which makes it an arduous task to compare them. Therefore, considering these analytical limitations, we developed a systematic review admitting its intrinsic qualitative nature by describing important points of bias, and we hope to contribute to future studies on avoiding those elements of bias that impair the quality of evidence.

## 6. Conclusion

Despite these limitations, our results support that is very important to evaluate and understand the role of cytokines and growth factors during the wound healing process, once these molecules have specific and important functions for each phase of the process acting in different pathways. In our review, we observed that extracellular molecules act more negatively in the wound healing process when silenced compared to intracellular and membrane molecules and the metabolic pathway more studied in the cutaneous process was TGF-*β*/Smad, and emphasis was given to the importance of the participation of macrophages in TGF-*β* signaling. In addition, the main molecules studied in knockout models in the healing process were the inflammatory cytokines and growth factors TGF-*β*1, VEGF, IL-6, TNF-*α*, and IL-1*β* and, consequently, the most investigated wound healing mechanisms were inflammation, angiogenesis, and consequently granulation tissue formation and collagen deposition. Besides that, some studies do not mention the participation of the molecules of gene depletion in metabolic pathways, which hinders the understanding of their role in wound healing mechanism. By using the right methodologies with the minimum risk of bias, we will be closer to finding specific biomarkers for wound healing. Overall, our findings provide new insights into the mechanisms of gene depletion in the wound healing process. However, the fragility of the current studies was evident, given that the majority presented unclear results, which can prevent the reproducibility of most of the studies considered in this review.

## Figures and Tables

**Table 1:** Main results of the gene depletion on immunoregulatory molecules involved with wound healing of knockout mice compared to the wild type.

References	Gene depletion	Predicted location	WH	Proinflammatory function	Anti-inflammatory function	Both function	Healing mechanisms
Ortega et al. (1998) [35]	*bFGF*	EC/I	D	Ø bFGF			(D) crust thickness, reepithelization
Lin et al. (2003) [26]	*IL-6*	EC/I	D	↓IL-1*α*, IL-1*β*, VEGF		↓TGF-*β*1	↓Neutrophil, macrophage, collagen production, angiogenesis = fibrinogen, platelet
Ishida et al. (2004) [41]	*IFN-γ*	EC	A	= IL-12, IL-18 ↑VEGF		↑TGF-*β*1	↓Macrophages ↑angiogenesis, collagen deposition
Guo et al. (1996) [38]	*KGF*	EC/ I	U	Ø KGF, = aFGF, bFGF, EGF, TNF-*α*			(U) reepithelization
Fang et al. (2007) [51]	*GM-CSF*	EC	D			↓IL − 6 = level of constitutive expression	↑(D) collagen deposition ↓acute inflammatory response = microvessel number
Yamauchi et al. (2016) [44]	*α-kl*	EC/M	D	↑IL-1*β*, TNF-*α*		↑IL-6	↓Collagen fibers, formation of the granulation tissue
Thuraisingam et al. (2006) [50]	*Nramp1*	M	D			↑TGF-*β*	—
Cogliati et al. (2015) [48]	*Cx43*	M	A			↑TGF-*β*1	= Collagen deposition ↑proliferation and activation of dermal fibroblasts
Braun et al. (2002) [56]	*Nrf2*	I	U	↓IL-1*β*, TNF-*α*		↓IL-6	(D) EC molecules
Thuraisingam et al. (2010) [49]	*MK2*	I	D	↓GM-CSF, VEGF, TNF, IL-1*β*		↓IL-6, IFN-*γ*	↓Angiogenesis, collagen deposition = neutrophils and macrophages
Somanath et al. (2008) [40]	*Akt1*	I	U	↓VEGF			↓Vascularization, collagen fibers, macrophages
Ashcroft et al. (1999) [39]	*Smad3*	I	A			↓TGF-*β*1	(A) reepithelization, ↓fibroblasts, and inflammatory cells
Kopecki et al. (2007) [57]	*c-Myb*	I	D			↑TGF-*β*1	↓Collagen deposition, cell proliferation
Wang et al. (2017) [28]	*P311*	I	D	↓VEGF		↓TGF-*β*1	↓Neoangiogenesis, tissue granulation remodeling, neocapillaries, reepithelization, length of the neoepithelium
Cheng et al. (2017) [27]	*P311*	I	U	↓TGF-*β*2	↓TGF-*β*3	↓TGF-*β*1	↓Collagen deposition, stiffness, and tensile strength in scars
Agah et al. (2002) [36]	*TSP1* and *TSP2*	EC	*TSP1* = D *TSP2* = A			TSP1 = ↓TGF-*β*1 TSP2 = TGF-*β*1	TPS1 = ↓collagen fibers, macrophages = vascularization TPS2 = ↓organization collagen fibers, matrix metalloproteinase expression ↑neovascularization
MacLauchlan et al. (2009) [37]	*TSP2*	EC	A	↑VEGF			= Collagen fibers, tensile strength, proliferation, myofibroblast differentiation
Gutiérrez-Fernández et al. (2007) [54]	*MMP8*	EC/I	D			↓TGF-*β*1	(D) reepithelization
Hattori et al. (2009) [42]	*MMP9* and *MMP13*	EC	*MMP9* = D *MMP13* = D	MMP9 = ↓intact CTGF MMP13 = ↑intact CTGF			(D) reepithelization = infiltration of inflammatory cells
Yeh et al. (2010) [55]	*Lum*	EC	D			↑TGF-*β*1 just was at day 6	↑Macrophages, inflammatory cells ↓skin thickness
Zhang et al. (2012) [52]	*Mstn*	EC	D			↓TGF-*β*	(D) reepithelization and wound contraction ↓collagen deposition, fibroblast-to-myofibroblast transformation
Zigrino et al. (2012) [47]	*MMP14*	I/M	D	= VEGF			= Formation of the granulation tissue, epidermal differentiation, formation ↑vascularization
Maeda et al. (2011) [43]	*ICOS* and *ICOSL*	*ICOS* = M *ICOSL* = I/M	*ICOS* = D *ICOSL* = D	↓TNF-*α*, CTGF = PDGF, VEGF	↓IL-4, IL-10	↓IL-6 = TGF-*β*, IFN-*γ*	↓Granulation tissue formation, vascularization, myofibroblasts proliferation, neutrophils, and macrophages infiltration
Kümin et al. (2007) [45]	*Prdx6*	I	U	= VEGF, IL-1*β*		= TGF-*β*1	= Area of hyperproliferative
Lundvig et al. (2014) [53]	*HO-2*	EC/I/M	D	= TNF	= Ang-1	= Ang-2	↓Collagen deposition
Guimarães et al. (2018) [29]	*5-LO*	I	A	↓TNF-*α*	= IL-10	↓TGF-*β*	↓Inflammatory infiltrate, mast cells, lymphocytes, matrix metalloproteinase expression ↑collagen deposition
Eming et al. (2007) [46]	*IL-10*	EC/I	A	↑VEGF-A-expressing mononuclear cells			(A) reepithelization ↑angiogenesis, myofibroblast differentiation, macrophages infiltration, collagen deposition ↓breakdown force

EC: extracellular; M: membrane; I: intracellular; WH: wound healing; ↑: increase; ↓: reduction; =: similar; Ø: absence: (D): delayed; (A): accelerated; (U): unchanged. 5-LO: 5-Lipoxygenase; aFGF: acid Fibroblastic Growth Factor; *α*-kl: alpha-Klotho; (Ang-1, Ang-2): Angiopoietin; bFGF: basic Fibroblastic Growth Factor; CTGF: Connective Tissue Growth Factor; Cx43: Connexin 43; EGF: Epidermal Growth Factor; GM-CSF: Granulocyte-Macrophage Colony-Stimulating Factor; HO-2: Heme Oxygenase 2; ICOS: Inducible Costimulator; ICOSL: Inducible Costimulator Ligand; IFN-*γ*: Interferon-gama; (IL-10, IL-6, IL-4, IL-12, IL-18, IL-1*β*, IL-1*α*): interleukins; KGF: Keratinocyte Growth Factor; Lum: Lumican; (MMP8, MMP9, MMP13, MMP14): Matrix Metalloproteinase; MK2: Mitogen-Activated Protein Kinase-2; Mstn: Myostatin; Nramp: Natural resistance-associated macrophage proteins; P311: Neuronal protein 3.1; Prdx6: Peroxiredoxin 6; PDGF: Platelet-Derived Growth Factor; Akt1: Serine/threonine kinase; (TPS1, TPS2): Thrombospondin; Nrf2: Transcription factor NF-E2-related factor 2; c-Myb: Transcription factor proto-oncogene c-Myb; (TGF-*β*, TGF-*β*1, TGF-*β*2, TGF-*β*3): Transforming Growth Factor; (TNF, TNF-*α*): Tumor Necrosis Factor; (VEGF, VEGF-A): Vascular Endothelial Growth Factor.

## Data Availability

The data can be made available upon request through the email: barbaracfn28@gmail.com.

## Supplementary Materials

Supplementary Materials Table S1: complete search strategy with search filters and the number of studies recovered in databases PubMed-Medline, Scopus, and Web of Science. Table S2: description of the main characteristics of studies of this systematic review that evaluated the effect of gene depletion on excision and incision wounds. Table S3: analysis of methodological bias of the studies founded in this systematic review that evaluated the effect of gene depletion on excision and incision wounds.
