# Supplementary material for: What Is the Impact of Depletion of Immunoregulatory Genes on Wound Healing? A Systematic Review of Preclinical Evidence
Source: Oxid Med Cell Longev. 2020 Dec 7;2020:8862953. doi: 10.1155/2020/8862953 (PMC7787779; doi:10.1155/2020/8862953)
Supplement: Supplementary Materials — Table S1: complete search strategy with search filters and the number of studies recovered in databases PubMed-Medline, Scopus, and Web of Science. Table S2: description of the main characteristics of studies of this systematic review that evaluated the effect of gene depletion on excision and incision wounds. Table S3: analysis of methodological bias of the studies founded in this systematic review that evaluated the effect of gene depletion on excision and incision wounds. [file 8862953.f1.docx]

| Table S1. Complete search strategy with search filters and number of studies recovered in databases PubMed/ Medline, Scopus and Web of Science. | | | |
| --- | --- | --- | --- |
| **PubMed/ Medline** | | | |
| **Descriptors** | **Items found** | **Date** | **Time** |
| Animals 1: #1  ("animal experimentation"[MeSH Terms] OR "models, animal"[MeSH Terms] OR "invertebrates"[MeSH Terms] OR "Animals"[Mesh:noexp] OR "animal population groups"[MeSH Terms] OR "chordata"[MeSH Terms:noexp] OR "chordata, nonvertebrate"[MeSH Terms] OR "vertebrates"[MeSH Terms:noexp] OR "amphibians"[MeSH Terms] OR "birds"[MeSH Terms] OR "fishes"[MeSH Terms] OR "reptiles"[MeSH Terms] OR "mammals"[MeSH Terms:noexp] OR "primates"[MeSH Terms:noexp] OR "artiodactyla"[MeSH Terms] OR "carnivora"[MeSH Terms] OR "cetacea"[MeSH Terms] OR "chiroptera"[MeSH Terms] OR "elephants"[MeSH Terms] OR "hyraxes"[MeSH Terms] OR "insectivora"[MeSH Terms] OR "lagomorpha"[MeSH Terms] OR "marsupialia"[MeSH Terms] OR "monotremata"[MeSH Terms] OR "perissodactyla"[MeSH Terms] OR "rodentia"[MeSH Terms] OR "scandentia"[MeSH Terms] OR "sirenia"[MeSH Terms] OR "xenarthra"[MeSH Terms] OR "haplorhini"[MeSH Terms:noexp] OR "strepsirhini"[MeSH Terms] OR "platyrrhini"[MeSH Terms] OR "tarsii"[MeSH Terms] OR "catarrhini"[MeSH Terms:noexp] OR "cercopithecidae"[MeSH Terms] OR "hylobatidae"[MeSH Terms] OR "hominidae"[MeSH Terms:noexp] OR "gorilla gorilla"[MeSH Terms] OR "pan paniscus"[MeSH Terms] OR "pan troglodytes"[MeSH Terms] OR "pongo pygmaeus"[MeSH Terms]) | 6529323 | 30/09/2019 | 03:14:26 |
| Animals 2: #2  (animals[TIAB] OR animal[TIAB] OR mice[TIAB] OR mus[TIAB] OR mouse[TIAB] OR murine[TIAB] OR woodmouse[TIAB] OR rats[TIAB] OR rat[TIAB] OR murinae[TIAB] OR muridae[TIAB] OR cottonrat[TIAB] OR cottonrats[TIAB] OR hamster[TIAB] OR hamsters[TIAB] OR cricetinae[TIAB] OR rodentia[TIAB] OR rodent[TIAB] OR rodents[TIAB] OR pigs[TIAB] OR pig[TIAB] OR swine[TIAB] OR swines[TIAB] OR piglets[TIAB] OR piglet[TIAB] OR boar[TIAB] OR boars[TIAB] OR "sus scrofa"[TIAB] OR ferrets[TIAB] OR ferret[TIAB] OR polecat[TIAB] OR polecats[TIAB] OR "mustela putorius"[TIAB] OR "guinea pigs"[TIAB] OR "guinea pig"[TIAB] OR cavia[TIAB] OR callithrix[TIAB] OR marmoset[TIAB] OR marmosets[TIAB] OR cebuella[TIAB] OR hapale[TIAB] OR octodon[TIAB] OR chinchilla[TIAB] OR chinchillas[TIAB] OR gerbillinae[TIAB] OR gerbil[TIAB] OR gerbils[TIAB] OR jird[TIAB] OR jirds[TIAB] OR merione[TIAB] OR meriones[TIAB] OR rabbits[TIAB] OR rabbit[TIAB] OR hares[TIAB] OR hare[TIAB] OR diptera[TIAB] OR flies[TIAB] OR fly[TIAB] OR dipteral[TIAB] OR drosphila[TIAB] OR drosophilidae[TIAB] OR cats[TIAB] OR cat[TIAB] OR carus[TIAB] OR felis[TIAB] OR nematoda[TIAB] OR nematode[TIAB] OR nematoda[TIAB] OR nematode[TIAB] OR nematodes[TIAB] OR sipunculida[TIAB] OR dogs[TIAB] OR dog[TIAB] OR canine[TIAB] OR canines[TIAB] OR canis[TIAB] OR sheep[TIAB] OR sheeps[TIAB] OR mouflon[TIAB] OR mouflons[TIAB] OR ovis[TIAB] OR goats[TIAB] OR goat[TIAB] OR capra[TIAB] OR capras[TIAB] OR rupicapra[TIAB] OR chamois[TIAB] OR haplorhini[TIAB] OR monkey[TIAB] OR monkeys[TIAB] OR anthropoidea[TIAB] OR anthropoids[TIAB] OR saguinus[TIAB] OR tamarin[TIAB] OR tamarins[TIAB] OR leontopithecus[TIAB] OR hominidae[TIAB] OR ape[TIAB] OR apes[TIAB] OR pan[TIAB] OR paniscus[TIAB] OR "pan paniscus"[TIAB] OR bonobo[TIAB] OR bonobos[TIAB] OR troglodytes[TIAB] OR "pan troglodytes"[TIAB] OR gibbon[TIAB] OR gibbons[TIAB] OR siamang[TIAB] OR siamangs[TIAB] OR nomascus[TIAB] OR symphalangus[TIAB] OR chimpanzee[TIAB] OR chimpanzees[TIAB] OR prosimians[TIAB] OR "bush baby"[TIAB] OR prosimian[TIAB] OR bush babies[TIAB] OR galagos[TIAB] OR galago[TIAB] OR pongidae[TIAB] OR gorilla[TIAB] OR gorillas[TIAB] OR pongo[TIAB] OR pygmaeus[TIAB] OR "pongo pygmaeus"[TIAB] OR orangutans[TIAB] OR pygmaeus[TIAB] OR lemur[TIAB] OR lemurs[TIAB] OR lemuridae[TIAB] OR horse[TIAB] OR horses[TIAB] OR pongo[TIAB] OR equus[TIAB] OR cow[TIAB] OR calf[TIAB] OR bull[TIAB] OR chicken[TIAB] OR chickens[TIAB] OR gallus[TIAB] OR quail[TIAB] OR bird[TIAB] OR birds[TIAB] OR quails[TIAB] OR poultry[TIAB] OR poultries[TIAB] OR fowl[TIAB] OR fowls[TIAB] OR reptile[TIAB] OR reptilia[TIAB] OR reptiles[TIAB] OR snakes[TIAB] OR snake[TIAB] OR lizard[TIAB] OR lizards[TIAB] OR alligator[TIAB] OR alligators[TIAB] OR crocodile[TIAB] OR crocodiles[TIAB] OR turtle[TIAB] OR turtles[TIAB] OR amphibian[TIAB] OR amphibians[TIAB] OR amphibia[TIAB] OR frog[TIAB] OR frogs[TIAB] OR bombina[TIAB] OR salientia[TIAB] OR toad[TIAB] OR toads[TIAB] OR "epidalea calamita"[TIAB] OR salamander[TIAB] OR salamanders[TIAB] OR eel[TIAB] OR eels[TIAB] OR fish[TIAB] OR fishes[TIAB] OR pisces[TIAB] OR catfish[TIAB] OR catfishes[TIAB] OR siluriformes[TIAB] OR arius[TIAB] OR heteropneustes[TIAB] OR sheatfish[TIAB] OR perch[TIAB] OR perches[TIAB] OR percidae[TIAB] OR perca[TIAB] OR trout[TIAB] OR trouts[TIAB] OR char[TIAB] OR chars[TIAB] OR salvelinus[TIAB] OR "fathead minnow"[TIAB] OR minnow[TIAB] OR cyprinidae[TIAB] OR carps[TIAB] OR carp[TIAB] OR zebrafish[TIAB] OR zebrafishes[TIAB] OR goldfish[TIAB] OR goldfishes[TIAB] OR guppy[TIAB] OR guppies[TIAB] OR chub[TIAB] OR chubs[TIAB] OR tinca[TIAB] OR barbels[TIAB] OR barbus[TIAB] OR pimephales[TIAB] OR promelas[TIAB] OR "poecilia reticulata"[TIAB] OR mullet[TIAB] OR mullets[TIAB] OR seahorse[TIAB] OR seahorses[TIAB] OR mugil curema[TIAB] OR atlantic cod[TIAB] OR shark[TIAB] OR sharks[TIAB] OR catshark[TIAB] OR anguilla[TIAB] OR salmonid[TIAB] OR salmonids[TIAB] OR whitefish[TIAB] OR whitefishes[TIAB] OR salmon[TIAB] OR salmons[TIAB] OR sole[TIAB] OR solea[TIAB] OR "sea lamprey"[TIAB] OR lamprey[TIAB] OR lampreys[TIAB] OR pumpkinseed[TIAB] OR sunfish[TIAB] OR sunfishes[TIAB] OR tilapia[TIAB] OR tilapias[TIAB] OR turbot[TIAB] OR turbots[TIAB] OR flatfish[TIAB] OR flatfishes[TIAB] OR sciuridae[TIAB] OR squirrel[TIAB] OR squirrels[TIAB] OR chipmunk[TIAB] OR chipmunks[TIAB] OR suslik[TIAB] OR susliks[TIAB] OR vole[TIAB] OR voles[TIAB] OR lemming[TIAB] OR lemmings[TIAB] OR muskrat[TIAB] OR muskrats[TIAB] OR lemmus[TIAB] OR otter[TIAB] OR otters[TIAB] OR marten[TIAB] OR martens[TIAB] OR martes[TIAB] OR weasel[TIAB] OR badger[TIAB] OR badgers[TIAB] OR ermine[TIAB] OR mink[TIAB] OR minks[TIAB] OR sable[TIAB] OR sables[TIAB] OR gulo[TIAB] OR gulos[TIAB] OR wolverine[TIAB] OR wolverines[TIAB] OR minks[TIAB] OR mustela[TIAB] OR llama[TIAB] OR llamas[TIAB] OR alpaca[TIAB] OR alpacas[TIAB] OR camelid[TIAB] OR camelids[TIAB] OR guanaco[TIAB] OR guanacos[TIAB] OR chiroptera[TIAB] OR chiropteras[TIAB] OR bat[TIAB] OR bats[TIAB] OR fox[TIAB] OR foxes[TIAB] OR iguana[TIAB] OR iguanas[TIAB] OR xenopus laevis[TIAB] OR parakeet[TIAB] OR parakeets[TIAB] OR parrot[TIAB] OR parrots[TIAB] OR donkey[TIAB] OR donkeys[TIAB] OR mule[TIAB] OR mules[TIAB] OR zebra[TIAB] OR zebras[TIAB] OR shrew[TIAB] OR shrews[TIAB] OR bison[TIAB] OR bisons[TIAB] OR buffalo[TIAB] OR buffaloes[TIAB] OR deer[TIAB] OR deers[TIAB] OR bear[TIAB] OR bears[TIAB] OR panda[TIAB] OR pandas[TIAB] OR "wild hog"[TIAB] OR "wild boar"[TIAB] OR fitchew[TIAB] OR fitch[TIAB] OR beaver[TIAB] OR beavers[TIAB] OR jerboa[TIAB] OR jerboas[TIAB] OR capybara[TIAB] OR capybaras[TIAB]) NOT medline[subset]) | 354824 | 30/09/2019 | 03:15:45 |
| Wound healing: #3  ("wound healing"[MeSH Terms] OR "regeneration"[MeSH Terms] OR "regeneration"[TIAB] OR "wound healing"[TIAB] OR "skin repair"[TIAB] OR "cutaneous repair"[TIAB] OR "skin healing"[TIAB] OR "cutaneous healing"[TIAB]) | 318717 | 30/09/2019 | 03:14:00 |
| Skin: #4  (“skin”[MeSH Terms] OR “dermis”[MeSH Terms] OR “epidermis”[MeSH Terms] OR "subcutaneous tissue"[MeSH Terms] OR “granulation tissue”[MeSH Terms] OR “keratinocytes”[MeSH Terms] OR "fibroblasts”[MeSH Terms] OR “integumentary system”[MeSH Terms] OR “skin”[TIAB] OR “dermis”[TIAB] OR “epidermis”[TIAB] OR "subcutaneous tissue"[TIAB] OR "hypodermis"[TIAB] OR “granulation tissue”[TIAB] OR “keratinocytes”[TIAB] OR "fibroblasts”[TIAB] OR “integumentary system”[TIAB] OR "skin injuries"[TIAB] OR "skin fibrosis"[TIAB] OR "skin scars[TIAB]) | 917631 | 30/09/2019 | 03:15:56 |
| (((#1) OR #2) AND #3) AND #4) | 1139 | 30/09/2019 | 03:16:27 |
| (((#1) OR #2) AND #3) AND #4) Filters: Portuguese; Spanish; English | 1130 | 30/09/2019 | 03:16:53 |
| **Scopus** | | | |
| **Descriptors** | **Items found** | **Date** | **Time** |
| Wound healing: #1  ( “wound healing” OR “regeneration” OR “skin repair” OR “cutaneous repair” OR “skin healing” OR “cutaneous healing” ) | 452808 | 30/09/2019 | 04:26:10 |
| Skin: #2  ( “skin” OR “dermis” OR “epidermis” OR “subcutaneous tissue” OR “granulation tissue” OR “keratinocytes” OR “fibroblasts” OR “integumentary system” OR “skin injuries” OR “skin fibrosis” OR “skin scars” ) | 1532971 | 30/09/2019 | 04:27:00 |
| #1 AND #2 | 1431 | 30/09/2019 | 04:29:15 |
| (#1 AND #2) ( LIMIT-TO ( LANGUAGE , AND , “English” , “Portuguese” , “Spanish” ) ) | 850 | 30/09/2019 | 04:30:17 |
| **Web of Science** | | | |
| **Descriptors** | **Items found** | **Date** | **Time** |
| Animals 1: #1  TS=("animal experimentation" OR “models, animal" OR “invertebrates" OR “Animals" OR “animal population groups" OR “chordata" OR “chordata, nonvertebrate" OR “vertebrates" OR “amphibians" OR “birds" OR “fishes" OR “reptiles" OR “mammals" OR “primates" OR “artiodactyla" OR “carnivora" OR “cetacea" OR “chiroptera" OR “elephants" OR “hyraxes" OR “insectivora" OR “lagomorpha" OR “marsupialia" OR “monotremata" OR “perissodactyla" OR “rodentia" OR “scandentia" OR “sirenia" OR “xenarthra" OR “haplorhini" OR “strepsirhini" OR “platyrrhini" OR “tarsii" OR “catarrhini" OR “cercopithecidae" OR “hylobatidae" OR “hominidae" OR “gorilla gorilla" OR “pan paniscus" OR “pan troglodytes" OR “pongo pygmaeus") | 1078578 | 30/09/2019 | 04:39:44 |
| Animals 2: #2  TS=(animals OR animal OR mice OR mus OR mouse OR murine OR woodmouse OR rats OR rat OR murinae OR muridae OR cottonrat OR cottonrats OR hamster OR hamsters OR cricetinae OR rodentia OR rodent OR rodents OR pigs OR pig OR swine OR swines OR piglets OR piglet OR boar OR boars OR "sus scrofa" OR ferrets OR ferret OR polecat OR polecats OR "mustela putorius" OR "guinea pigs" OR "guinea pig" OR cavia OR callithrix OR marmoset OR marmosets OR cebuella OR hapale OR octodon OR chinchilla OR chinchillas OR gerbillinae OR gerbil OR gerbils OR jird OR jirds OR merione OR meriones OR rabbits OR rabbit OR hares OR hare OR diptera OR flies OR fly OR dipteral OR drosphila OR drosophilidae OR cats OR cat OR carus OR felis OR nematoda OR nematode OR nematoda OR nematode OR nematodes OR sipunculida OR dogs OR dog OR canine OR canines OR canis OR sheep OR sheeps OR mouflon OR mouflons OR ovis OR goats OR goat OR capra OR capras OR rupicapra OR chamois OR haplorhini OR monkey OR monkeys OR anthropoidea OR anthropoids OR saguinus OR tamarin OR tamarins OR leontopithecus OR hominidae OR ape OR apes OR pan OR paniscus OR "pan paniscus" OR bonobo OR bonobos OR troglodytes OR "pan troglodytes" OR gibbon OR gibbons OR siamang OR siamangs OR nomascus OR symphalangus OR chimpanzee OR chimpanzees OR prosimians OR "bush baby" OR prosimian OR bush babies OR galagos OR galago OR pongidae OR gorilla OR gorillas OR pongo OR pygmaeus OR "pongo pygmaeus" OR orangutans OR pygmaeus OR lemur OR lemurs OR lemuridae OR horse OR horses OR pongo OR equus OR cow OR calf OR bull OR chicken OR chickens OR gallus OR quail OR bird OR birds OR quails OR poultry OR poultries OR fowl OR fowls OR reptile OR reptilia OR reptiles OR snakes OR snake OR lizard OR lizards OR alligatOR OR alligators OR crocodile OR crocodiles OR turtle OR turtles OR amphibian OR amphibians OR amphibia OR frog OR frogs OR bombina OR salientia OR toad OR toads OR "epidalea calamita" OR salamander OR salamanders OR eel OR eels OR fish OR fishes OR pisces OR catfish OR catfishes OR siluriformes OR arius OR heteropneustes OR sheatfish OR perch OR perches OR percidae OR perca OR trout OR trouts OR char OR chars OR salvelinus OR "fathead minnow" OR minnow OR cyprinidae OR carps OR carp OR zebrafish OR zebrafishes OR goldfish OR goldfishes OR guppy OR guppies OR chub OR chubs OR tinca OR barbels OR barbus OR pimephales OR promelas OR "poecilia reticulata" OR mullet OR mullets OR seahorse OR seahorses OR mugil curema OR atlantic cod OR shark OR sharks OR catshark OR anguilla OR salmonid OR salmonids OR whitefish OR whitefishes OR salmon OR salmons OR sole OR solea OR "sea lamprey" OR lamprey OR lampreys OR pumpkinseed OR sunfish OR sunfishes OR tilapia OR tilapias OR turbot OR turbots OR flatfish OR flatfishes OR sciuridae OR squirrel OR squirrels OR chipmunk OR chipmunks OR suslik OR susliks OR vole OR voles OR lemming OR lemmings OR muskrat OR muskrats OR lemmus OR otter OR otters OR marten OR martens OR martes OR weasel OR badger OR badgers OR ermine OR mink OR minks OR sable OR sables OR gulo OR gulos OR wolverine OR wolverines OR minks OR mustela OR llama OR llamas OR alpaca OR alpacas OR camelid OR camelids OR guanaco OR guanacos OR chiroptera OR chiropteras OR bat OR bats OR fox OR foxes OR iguana OR iguanas OR xenopus laevis OR parakeet OR parakeets OR parrot OR parrots OR donkey OR donkeys OR mule OR mules OR zebra OR zebras OR shrew OR shrews OR bison OR bisons OR buffalo OR buffaloes OR deer OR deers OR bear OR bears OR panda OR pandas OR "wild hog" OR "wild boar" OR fitchew OR fitch OR beaver OR beavers OR jerboa OR jerboas OR capybara OR capybaras) | 7217976 | 30/09/2019 | 04:39:55 |
| Wound healing: #3  TS=("wound healing" OR “regeneration" OR “regeneration" OR “wound healing" OR “skin repair" OR “cutaneous repair" OR “skin healing" OR “cutaneous healing") | 298843 | 30/09/2019 | 04:39:20 |
| Skin: #4  TS=(“skin” OR “dermis” OR “epidermis” OR “subcutaneous tissue" OR “granulation tissue” OR “keratinocytes” OR “fibroblasts” OR “integumentary system” OR “skin” OR “dermis” OR “epidermis” OR “subcutaneous tissue" OR “hypodermis" OR “granulation tissue” OR “keratinocytes” OR “fibroblasts” OR “integumentary system” OR “skin injuries" OR “skin fibrosis" OR “skin scars”) | 785327 | 30/09/2019 | 04:40:20 |
| (((#1) OR #2) AND #3) AND #4) | 300 | 30/09/2019 | 04:40:25 |
| (((#1) OR #2) AND #3) AND #4) AND IDIOMA: (English OR Portuguese OR Spanish) | 297 | 30/09/2019 | 04:40:33 |

Table S2. Description of main characteristics of studies of this systematic review that evaluated the effect of gene depletion on excision and incision wounds.

| Ref. | Co. | Strain | KO | S | Age | W | Antis. | Anesthesia | Inst. for biopsy | Wound N/Size | Biopsy (days) | WH (days) | Material | Methods |  |
| --- | --- | --- | --- | --- | --- | --- | --- | --- | --- | --- | --- | --- | --- | --- | --- |
| **Excisional wounds** | | | | | | | | | | | | | | | |
| Ortega et al. (1998) [33] | USA | C57BL/6 | *bFGF* | ? | 8-11 w | ? | 70% Alcohol | Avertin | Curved scissors | 1/ ø6 mm | ? | 0, 6, 10, 12, 14, 16, 17, 18, 20 | Extracted protein | WB |  |
| Agah et al. (2002)  [34] | USA | 129SvJ and 129SvTer | *TSP1* and *TSP2* | ? | 3 m | ? | ? | ? | ? | ?/ ø6 mm | ? | ? | Extracted protein | ELISA |  |
| Braun et al. (2002) [35] | SWI | BALB/c | *Nrf2* | ? | 8-12 w | ? | ? | Kx | ? | 2/ ø5 mm | 1, 5 and 13 | ? | Extracted protein and Extracted RNA | RPA and ELISA |  |
| Lin et al. (2003) [26] | JPN | BALB/c | *IL-6* | M | 8 w | ? | 70% Alcohol | Pent. | Biopsy punch | 6/ ø4 mm | 1, 3, 6, 10 and 14 | 1, 3, 6, 10 and 14 | Extracted RNA | RT-PCR |  |
| Ishida et al. (2004) [36] | JPN | BALB/c | *IFN-γ* | M | 8-12 w | ? | 70% Alcohol | Pent. | Biopsy punch | 6/ ø4 mm | 1, 3, 6, 10 and 14 | 1, 3, 6, 10 and 14 | Extracted RNA | RT-PCR and Western blot |  |
| Thuraisingam et al. (2006) [37] | CAN | 129J | *Nramp1* | ? | 8-10 w | ? | 70% Alcohol | Isoflurane inhalation | Biopsy punch | 1/ ø6 mm | ? | 0, 2, 4, 6, 8, 10, 12 and 14 | Tissues in paraffin | IHC |  |
| Kümin et al. (2007) [38] | GER | ? | *Prdx6* | ? | ? | ? | ? | Kx | ? | 2/ ø5 mm | ? | ? | Extracted RNA | RPA |  |
| Eming et al. (2007) [39] | GER | C57BL/6 | *IL-10* | M | 10-12 w | ? | ? | Kx | Biopsy punch | 4/ ø6 mm | ? | 1, 3, 5, 7, 9, 11, 13 and 15 | Tissues in paraffin | Immunofluorescent staining |  |
| Fang et al. (2007) [40] | CHN | C57BL/6 | *GM-CSF* | ? | 8-10 w | 19-22 g | Povidone-iodine and 70%  Alcohol | Avertin | ? | ?/ 0.64 cm² | 1, 3, 5, 7, 10 and 14 | 0, 2, 3, 4, 5, 6, 7, 8, 10, 12 and 14 | Extracted protein | ELISA |  |
| MacLauchlan et al. (2009) [41] | USA | 129SvJ | *TSP2* | ? | 3 m | ? | ? | Avertin | Biopsy punch | 2/ ø6 mm | ? | ? | Extracted protein | WB and ELISA |  |
| Gutiérrez - Fernández et al. (2007) [42] | SPA | C57BL/6J/129 | *MMP8* | M | 8-12 w | ? | 70% Alcohol | Isoflurane inhalation | Biopsy punch | 2/ ø8 mm | ? | 1, 3, 5, 7, 9, 11 and 13 | Extracted protein | ELISA |  |
| Hattori et al. (2009) [43] | JPN | 129SvEv/CD-1 for MMP9; 129/Sv and C57BL/6J for MMP13 | *MMP9* and *MMP13* | ? | 9-11 w | ? | ? | ? | ? | ?/ ø8 mm | ? | 0, 1, 3, 5, 7 and 10 | Separate protein | Densitometrical analysis and IB |  |
| Thuraisingam et al. (2010) [44] | CAN | 129/J x C57BL/6 | *MK2* | F | 8-10 w | ? | ? | ? | ? | ?/ ? | ? | 0, 3, 6, 12, 18 | Extracted protein | Lincoplex  mouse cytokine kit |  |
| Yeh et al. (2010) [45] | TWN | C57BL/6 | *Lum* | M | 4-6 w | ? | Alcohol swab | Kx | Biopsy punch | 1/ ø6 mm | 3, 6, 12 and 18 | 0, 3, 6, 12 and 18 | Extracted RNA | Semiquantitative RT-PCR |  |
| Maeda et al. (2011) [46] | JPN | C57BL/6 | *ICOS* and *ICOSL* | M | 8-10 w | ? | ? | ? | ? | 4/ ø6 mm | ? | 0, 3, 5, 7, 10 and 15 | Extracted protein | RT-qPCR and CBA |  |
| Zhang et al. (2012) [47] | SIN | C57BL/6 | *Mstn* | F | 6 w | ? | ? | ? | ? | 2/ 0.25 cm² | 1, 3, 5, 7, 10, 12 and 15 | 0, 1, 3, 5, 7, 10, 12 and 15 | Extracted RNA | qPCR |  |
| Zigrino et al. (2012) [48] | GER | C57BL/6 | *MMP14* | ? | 8 w | ? | ? | ? | ? | 2/ ø4 mm | ? | 1, 3, 5, 7, 9, 11 and 14 | Extracted RNA | WB and RT-PCR |  |
| Lundvig et al. (2014) [49] | NLD | 129Sv x C57BL/6 | *HO-2* | F | 6-12 w | ? | ? | ? | Biopsy punch | 4/ ø4 mm | ? | 0, 1, 2, 5 and 7 | Extracted RNA | qPCR |  |
| Cogliati et al. (2015) [50] | BR | CD1 | *Cx43* | M | 8 w | ±30 g | Iodine solution | Kx | Biopsy punch | 2/ ø5 mm | 3, 7 and 14 | 0, 1, 3, 5, 7, 9 and 11 | Extracted RNA | qPCR |  |
| Yamauchi et al. (2016) [51] | JPN | ? | *α-kl* | ? | 5-6 w | ? | ? | Isoflurane inhalation | Biopsy punch | 1/ ø6 mm | 3, 6, 8, 10 and 12 | 0, 1, 3, 5, 7, 9, 11, 13, 15, 17, 19 and 21 | Extracted RNA | RT-PCR |  |
| Cheng et al. (2017) [27] | USA | C57BL/6 | *P311* | M | 4 m | ? | 75% Alcohol | Kx | Biopsy punch | 1/ ø6 mm | 14 and 21 | 0, 3, 7, 14 e 21 | Extracted RNA | ELISA |  |
| Wang et al. (2017) [28] | CHN | C57BL/6NCr1Br × 129S1/SvImJ | *P311* | M | 12 w | ? | ? | Pent. | Biopsy punch | 2/ ø4 mm | ? | 0, 3, 5 and 7 | Extracted protein | WB and ELISA |  |
| Guimarães et al. (2018) [29] | BR | 129-Alox5tm1Fun | *5-LO* | F | 6-8 w | ? | ? | ? | ? | ?/ 19 mm² | 1, 2, 5 and 10 | 0, 1, 2, 3, 4, 5, 6, 7, 8, 9 and 10 | Extracted RNA | RT-qPCR |  |
| **Incisional wounds** | | | | | | | | | | | | | | | |
| Guo et al. (1996) [52] | USA | ? | *KGF* | ? | ? | ? | ? | ? | Scalpel | ?/? | ? | 1, 3, 5 and 7 | Extracted RNA | RT-PCR |  |
| Ashcroft et al. (1999) [53] | USA | ? | *Smad3* | ? | 4-6 w | ? | Alcohol | Methoxyfluorane | ? | 4/ 1 cm | 1, 2, 3, 5 | 1, 2, 3 and 5 | Tissues in paraffin and Extracted RNA | IHC and RPA |  |
| Kopecki et al. (2007) [54] | AUS | BALB/c | *c-Myb* | ? | 16-20 w | ? | ? | ? | ? | 2/ 1 cm | ? | 0, 3, 7, and 14 | Tissues of histology and Extracted RNA | IHC and RT-qPCR |  |
| Somanath et al. (2008) [55] | USA | 129 R1/  C57BL/6 | *Akt1* | ? | 8-12 w | ? | 30% Isopropyl alcohol | Kx | ? | ?/ 15 mm | 4, 7 | 1, 2, 3, 4, 5, 6 and 7 | Extracted protein | ELISA |  |

References (Ref.); Country (Co); Sex (S); Weight (W); Antisepsis (Antis.); Instrument for biopsy (Inst. for biopsy); No information/ unclear (?); Knockout (KO); Number of wounds (N); weeks (w); months (m); diameter (ø); Male (M); Female (F); grams (g); Ketamine and xylazine (Kx); Pentobarbital (Pent.). Enzyme-Linked Immunosorbent Assay (ELISA); Reverse-Transcriptase Polymerase Chain Reaction (RT-PCR); Real-Time quantitative Polymerase Chain Reaction (qPCR); Reverse-transcription real-time Polymerase Chain Reaction (RT-qPCR); Immunohistochemistry (IHC); Cytometric Bead Array (CBA); Immunoblotting (IB); Western Blot (WB); RNase Protection Assay (RPA). United States of America (USA), Australia (AUS), Brazil (BR), China (CHN), Japan (JPN), Germany (GER), Spain (SPA), Canada (CAN), Taiwan (TWN), Switzerland (SWI), Netherlands (NLD), Singapore (SIN). basic Fibroblastic Growth Factor (bFGF), Thrombospondin (TPS1, TPS2), Transcription factor NF-E2-related factor 2 (Nrf2), Interleukins (IL-6, IL-10), Interferon-gamma (IFN-γ), Natural resistance-associated macrophage proteins (Nramp), Peroxiredoxin 6 (Prdx6), Granulocyte-Macrophage Colony-Stimulating Factor (GM-CSF), Matrix Metalloproteinase (MMP8, MMP9, MMP13, MMP14), Mitogen-Activated Protein Kinase-2 (MK2), Lumican (Lum), Inducible Costimulator (ICOS), Inducible Costimulator Ligand (ICOSL), Myostatin (Mstn), Heme oxygenase 2 (HO-2), Connexin 43 (Cx43), alpha-klotho (α-kl), Neuronal protein 3.1 (P311), 5-Lipoxygenase (5-LO), Keratinocyte Growth Factor (KGF), Transcription factor proto-oncogene c-Myb (c-Myb), Serine/threonine kinase (Akt1). The days indicated for "Biopsy (days)" corresponds to the days on which materials were collected for histological and immunoregulatory molecule analyzes, while the days indicated "WH (days)" correspond to these analyzes including the macroscopic analysis that corresponds to the monitoring of the closure of the cutaneous wound directly from the observation of the animal.

Table S3. Analysis of methodological bias of the studies founded in this systematic review that evaluated the effect of gene depletion on excision and incision wounds.

|  | Guo et al. (1996) [52] | Ortega et al. (1998) [33] | Ashcroft et al. (1999) [53] | Agah et al. (2002) [34] | Braun et al. (2002) [35] | Lin et al. (2003) [26] | Ishida et al. (2004) [36] | Thuraisingam et al. (2006) [37] | Kümin et al. (2007) [38] | Eming et al. (2007) [39] | Fang et al. (2007) [40] | Gutiérrez - Fernández et al. (2007) [42] | Kopecki et al. (2007) [54] | Somanath et al. (2008) [55] | Hattori et al. (2009) [43] | MacLauchlan et al. (2009) [41] | Thuraisingam et al. (2010) [44] | Yeh et al. (2010) [45] | Maeda et al. (2011) [46] | Zhang et al. (2012) [47] | Zigrino et al. (2012) [48] | Lundvig et al. (2014) [49] | Cogliati et al. (2015) [50] | Yamauchi et al. (2016) [51] | Cheng et al. (2017) [27] | Wang et al. (2017) [28] | Guimarães et al. (2018) [29] | Criteria completed (n) | Criteria completed (%) |
| --- | --- | --- | --- | --- | --- | --- | --- | --- | --- | --- | --- | --- | --- | --- | --- | --- | --- | --- | --- | --- | --- | --- | --- | --- | --- | --- | --- | --- | --- |
| 1 | X | X | X | X | X | X | X | X | X | X | X | X | X | X | X | X | X | X | X | X | X | X | X | X | X | X | X | 27 | 100.0 |
| 2 | X | X | X | X | X | X | X | X | X | X | X | X | X | X | X | X | X | X | X | X | X | X | X | X | X | X | X | 27 | 100.0 |
| 3 | X | X | X | X | X | X | X | X | X | X | X | X | X | X | X | X | X | X | X | X | X | X | X | X | X | X | X | 27 | 100.0 |
| 4 | X | X | X | X | X | X | X | X | X | X | X | X | X | X | X | X | X | X | X | X | X | X | X | X | X | X | X | 27 | 100.0 |
| 5 |  |  |  | X | X | X | X | X | X | X | X | X | X | X | X | X | X | X | X | X |  | X | X | X | X | X | X | 23 | 85.2 |
| 6 |  |  |  |  |  |  |  |  |  |  |  |  |  |  |  |  |  |  |  |  |  |  |  |  |  |  |  | 0 | 0 |
| 7 |  |  |  |  |  |  |  |  |  |  |  |  |  |  |  |  |  |  |  |  |  |  |  |  |  |  |  | 0 | 0 |
| 8 | X | X | X | X | X | X | X | X | X | X | X | X | X | X | X | X | X | X | X | X | X | X | X | X | X | X | X | 27 | 100.0 |
| 9 |  |  |  |  |  | X | X |  |  | X |  | X |  |  |  |  | X | X | X | X |  | X | X |  | X | X | X | 13 | 48.1 |
| 10 |  | X | X | X | X | X | X | X |  | X | X | X | X | X | X | X | X | X | X | X | X | X | X | X | X | X | X | 25 | 92.6 |
| 11 |  |  |  |  |  |  |  |  |  |  | X |  |  |  |  |  |  |  |  |  |  |  | X |  |  |  |  | 2 | 7.4 |
| 12 |  | X |  | X | X | X | X | X |  | X | X | X | X | X | X | X | X | X | X | X | X | X | X |  | X | X | X | 23 | 85.2 |
| 13 |  |  |  |  |  | X | X | X |  | X | X | X | X |  |  |  | X |  | X |  |  | X |  |  |  |  | X | 10 | 37.0 |
| 14 |  |  |  |  |  |  |  |  |  |  | X |  |  |  |  |  |  |  |  |  |  | X | X |  | X |  | X | 5 | 18.5 |
| 15 |  |  |  |  |  |  |  |  |  |  |  |  |  |  |  |  |  |  |  |  |  |  | X |  | X |  |  | 2 | 7.4 |
| 16 |  |  |  |  |  |  |  |  |  |  |  |  |  |  |  |  |  |  |  |  |  |  | X |  |  |  |  | 1 | 3.7 |
| 17 |  |  |  |  |  |  |  |  | X |  | X |  |  |  |  |  |  |  |  |  |  | X | X |  |  |  | X | 5 | 18.5 |
| 18 | X | X |  |  |  | X | X | X |  | X |  | X |  |  |  | X |  | X |  |  |  | X | X | X | X | X |  | 14 | 51.9 |
| 19 |  | X | X |  | X | X | X | X | X | X | X | X |  | X |  | X |  | X |  |  |  |  | X | X | X | X |  | 17 | 63.0 |
| 20 |  | X | X |  |  | X | X | X |  |  | X | X |  | X | X |  |  | X |  |  |  |  | X |  | X |  |  | 11 | 40.7 |
| 21 |  |  |  |  |  |  |  |  |  |  |  |  |  |  |  |  |  |  |  |  |  |  |  |  |  |  |  | 0 | 0 |
| 22 |  |  |  |  |  |  |  |  |  |  |  |  |  |  |  |  |  |  |  |  |  |  |  |  |  |  |  | 0 | 0 |
| 23 | X | X | X | X | X | X | X | X | X | X | X | X | X | X | X | X | X | X | X | X | X | X | X | X | X | X | X | 27 | 100.0 |
| 24 |  |  |  |  |  | X | X | X | X | X | X | X | X | X |  | X | X | X | X | X | X | X | X | X | X | X | X | 22 | 81.5 |
| 25 |  |  |  |  |  |  |  |  |  |  |  |  |  |  |  |  |  |  |  |  |  |  |  |  |  |  |  | 0 | 0 |
| 26 |  |  |  |  |  |  |  |  |  |  |  |  |  |  |  |  |  |  |  |  |  |  |  |  |  |  |  | 0 | 0 |
| 27 |  | X | X | X | X | X | X | X | X | X | X | X | X | X | X | X | X | X | X | X | X | X | X | X | X | X | X | 26 | 96.3 |
| 28 |  |  |  |  |  |  |  |  |  |  |  |  |  |  |  |  |  |  |  |  |  |  |  |  |  |  |  | 0 | 0 |
| 29 | X | X | X | X | X | X | X | X | X | X | X | X | X | X | X | X | X | X | X | X | X | X | X | X | X | X | X | 27 | 100.0 |
| 30 |  |  |  |  |  |  |  |  |  |  |  |  |  |  |  |  |  |  |  |  |  |  |  |  |  |  |  | 0 | 0 |
| 31 |  |  |  |  |  |  |  |  |  |  |  |  |  |  |  |  |  |  |  |  |  |  |  |  |  |  |  | 0 | 0 |
|  | 8 | 13 | 11 | 11 | 12 | 17 | 17 | 16 | 12 | 16 | 18 | 17 | 13 | 14 | 12 | 14 | 14 | 16 | 14 | 13 | 11 | 17 | 21 | 13 | 18 | 14 | 16 |  |  |

(1) Accurate and concise description of the article content, (2) Background summary, research objective, methods, principal findings, and conclusions, (3) Sufficient scientific background, (4) Explanation of the experimental approach and rationale, (5) Nature of the ethical review permissions, relevant licenses, and national or institutional guidelines for the care and use of animals, (6) Number of animals per group, (7) Randomization of the experiment and blind controlled study, (8) Experimental unit, (9) Animal sex, (10) Animal age, (11) Animal weight, (12) Animal strain, (13) Pathogen free housing, (14) Light/dark cycle, (15) Temperature, (16) Humidity, (17) Water and food, (18) Biopsy instrument, (19) Anesthesia, (20) Antisepsis, (21) Total number of animals, choice of sample size and repetitions, (22) Full details of how animals were allocated to experimental groups (including randomization or matching), (23) Experimental outcomes, (24) Statistical analysis, (25) Description of animals’ health status, for each experimental group, before treatment, (26) Number of animals in each group included in each analysis (including explanations of the non-inclusion of some animals), (27) Results of analysis carried out, with a measure of precision, (28) Modifications to the experimental protocols made to reduce adverse events, (29) Interpretation of the results, taking into account the study objectives and hypotheses, current theory, and relevant studies, (30) Comments on how the findings are likely to translate to other species, (31) List all funding sources and the role of the funder(s) in the study.
